# Targeting ESKAPE pathogens with anti-infective medicinal plants from the Greater Mpigi region in Uganda

**DOI:** 10.1038/s41598-020-67572-8

**Published:** 2020-07-20

**Authors:** Fabien Schultz, Godwin Anywar, Huaqiao Tang, François Chassagne, James T. Lyles, Leif-Alexander Garbe, Cassandra L. Quave

**Affiliations:** 1 Department of Dermatology, Emory University School of Medicine, 615 Michael St., Atlanta, GA 30322 USA; 2 Institute of Biotechnology, Faculty III - Process Sciences, Technical University of Berlin, Gustav-Meyer-Allee 25, 13355 Berlin, Germany; 3 Department of Agriculture and Food Sciences, Neubrandenburg University of Applied Sciences, Brodaer Str. 2, 17033 Neubrandenburg, Germany; 4 Department of Plant Sciences, Microbiology and Biotechnology, Makerere University, P.O. Box 7062, Kampala, Uganda; 5 ZELT - Neubrandenburg Center for Nutrition and Food Technology gGmbH, Seestraße 7A, 17033 Neubrandenburg, Germany; 6 Center for Study of Human Health, Emory University College of Arts and Sciences, 615 Michael St., Atlanta, GA 30322 USA; 7 Emory Antibiotic Resistance Center, Emory University, 615 Michael St., Atlanta, GA 30322 USA

**Keywords:** Antimicrobials, Plant sciences, Medical research

## Abstract

Antibiotic resistance poses one of the greatest threats to global health today; conventional drug therapies are becoming increasingly inefficacious and limited. We identified 16 medicinal plant species used by traditional healers for the treatment of infectious and inflammatory diseases in the Greater Mpigi region of Uganda. Extracts were evaluated for their ability to inhibit growth of clinical isolates of multidrug-resistant ESKAPE pathogens. Extracts were also screened for quorum quenching activity against *S. aureus*, including direct protein output assessment (δ-toxin), and cytotoxicity against human keratinocytes (HaCaT). Putative matches of compounds were elucidated via LC–FTMS for the best-performing extracts. These were extracts of *Zanthoxylum chalybeum* (*Staphylococcus aureus*: MIC: 16 μg/mL; *Enterococcus faecium*: MIC: 32 μg/mL) and *Harungana madagascariensis* (*S. aureus*: MIC: 32 μg/mL; *E. faecium*: MIC: 32 μg/mL) stem bark. Extracts of *Solanum aculeastrum* root bark and *Sesamum calycinum subsp. angustifolium* leaves exhibited strong quorum sensing inhibition activity against all *S. aureus* accessory gene regulator (*agr*) alleles in absence of growth inhibition (IC_50_ values: 1–64 μg/mL). The study provided scientific evidence for the potential therapeutic efficacy of these medicinal plants in the Greater Mpigi region used for infections and wounds, with 13 out of 16 species tested being validated with in vitro studies.

## Introduction

The rise of antimicrobial resistance (AMR) requires mobilization of political, financial and research investment due to its emergence as a global health hazard that threatens the ability to treat infectious diseases^1^. According to the World Health Organization, AMR poses “one of the biggest threats to global health, food security, and development today” and can affect anyone in any country and of any age^2^. Today, AMR already accounts for 700,000 deaths annually. By 2050, this figure is estimated to reach more than 10 million deaths per year, which is more people than currently die from cancer^3^. Because effective antibiotics are critical for treatment of bacterial infections and for procedures where there is a high risk of infection, e.g. surgery, new anti-infectives are needed to overcome this global threat^4^. The issue of resistance is not uniformly spread across all bacteria^5^. Six species have been identified by the Infectious Disease Society of America (IDSA) as being especially dangerous due to their potential multidrug resistance mechanisms and virulence. They are referred to as ‘ESKAPE’ pathogens, which is an acronym for *Enterococcus faecium*, *Staphylococcus aureus*, *Klebsiella pneumoniae*, *Acinetobacter baumannii*, *Pseudomonas aeruginosa* and *Enterobacter species.* This group of pathogenic bacteria encompasses both Gram-negative and Gram-positive species that are capable of ‘escaping’ bactericidal action of conventional antibiotics^6,7^. ESKAPE pathogens are common causes of deadly or life-threatening infections, especially among children, immunocompromised, and critically-ill people^8^.

Antibiotics are not the only anti-infectives that could provide an effective weapon against these pathogens. Another therapeutic, yet non-antibiotic, strategy is targeting bacterial virulence controlled by quorum sensing processes. The quorum-sensing mechanism mediated by signal molecules regulates the expression of virulence genes in the majority of pathogenic bacteria, meaning that quorum-sensing inhibitors are expected to be one of the best alternatives to antibiotics^9,10^. Autoinducers, self-secreted signal molecules, are regulated by a density-dependent synchronized gene expression system during quorum sensing^11^. Biofilm formation, toxin production and other virulence factors are controlled by quorum sensing and the production of virulence factors can weaken the balance of host defense mechanisms^9^. Initiation of toxin production occurs when extracellular signaling and communication indicates that a threshold population of bacteria has been achieved^12^. Inhibition of quorum sensing induced by secondary plant metabolites can significantly attenuate bacterial virulence and substantially enhance vulnerability to conventional antibiotics and to the immune system^9,12–14^.

It is estimated that more than 25% of the Western drugs prescribed contain plant-derived natural products as active ingredients^15^. Yet, only a small proportion of plant species has ever been investigated for pharmacological activity in a laboratory setting^16,17^. In East and Central Africa, medicinal plant use and traditional medicine practices are still the predominant form of healthcare^18,19^. In Uganda, four out of five patients primarily seek medical treatment from traditional healers instead of Western-trained physicians and there is at least one traditional healer per village practicing traditional use of medicinal plants^20,21^. Despite its small size, Uganda is characterized by its very rich biological diversity of 5,000 species of higher plants in the indigenous flora^22^, resulting from its unique bio-geographical location^23^. Documentation of traditional use and ethnopharmacological evaluation of this wealth of plant species can still be considered an understudied field.

**Figure 1:**
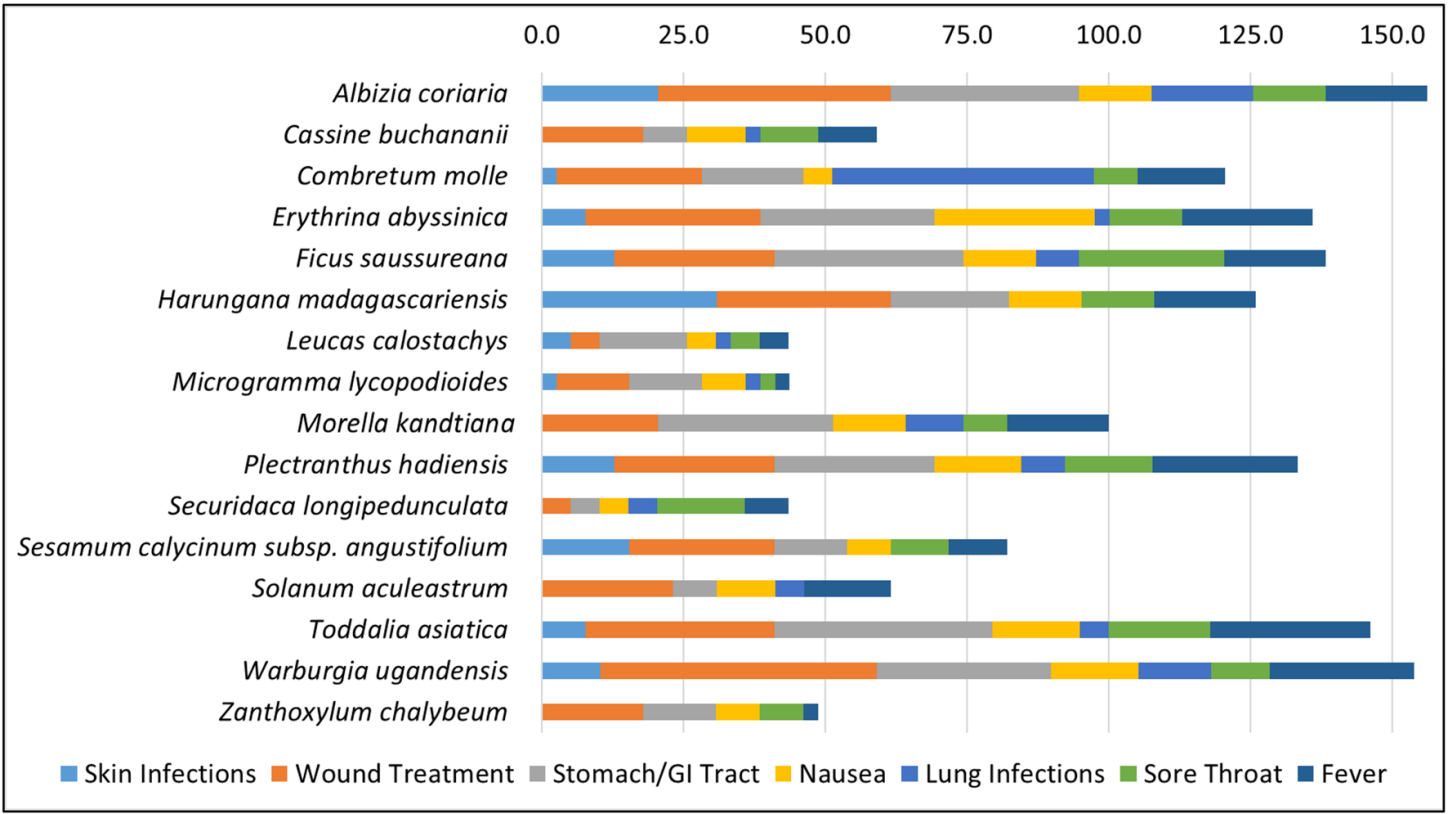
Ethnopharmacological information on the medicinal use of plant candidates from the Greater Mpigi region in Uganda (with emphasis on infections and symptoms of infections). The stacked histogram figure shows the relative frequencies of citation (RFC) in % in treatment of relevant medical disorders, calculated from data obtained through an ethnobotanical survey of 39 traditional healers. Here, the RFC assesses the importance of a plant species used for a specific medical condition relative to the total number of informants interviewed in the study. It varies from 0% (none of the informants uses this plant species in treatment of a specific medical condition) to 100% (maximum number of informants use this plant species in treatment of a specific medical condition)^24^. Consequently, the higher the value of cumulated RFCs (x-axis), the higher the traditional use of a plant species in treatment of medical conditions relevant to this study.

A recent ethnobotanical study by Schultz et al. identified 16 medicinal plant species that play a significant role in the local traditional medicine of the Greater Mpigi region located in West-Central Uganda^24^. The local vegetation at the study site is characterized as a tropical, moist evergreen forest/savanna mosaic^25,26^. Here, people are highly dependent on medicinal plants and local traditional healers for primary health care. Apart from many other traditional uses documented, 16 medicinal plants were found to be critical to anti-infective traditional medicine practices in the Greater Mpigi region (in particular, skin and wound infections, and symptoms associated with bacterial infections). The majority of the plant species have not been studied for potential bioactivity yet^24^. As the ethnopharmacological basis for this study, these species, their traditional use in treatment of infections and the relative frequency of citation in % (*n* = 39) are illustrated in Fig. 1.

**Figure 2:**
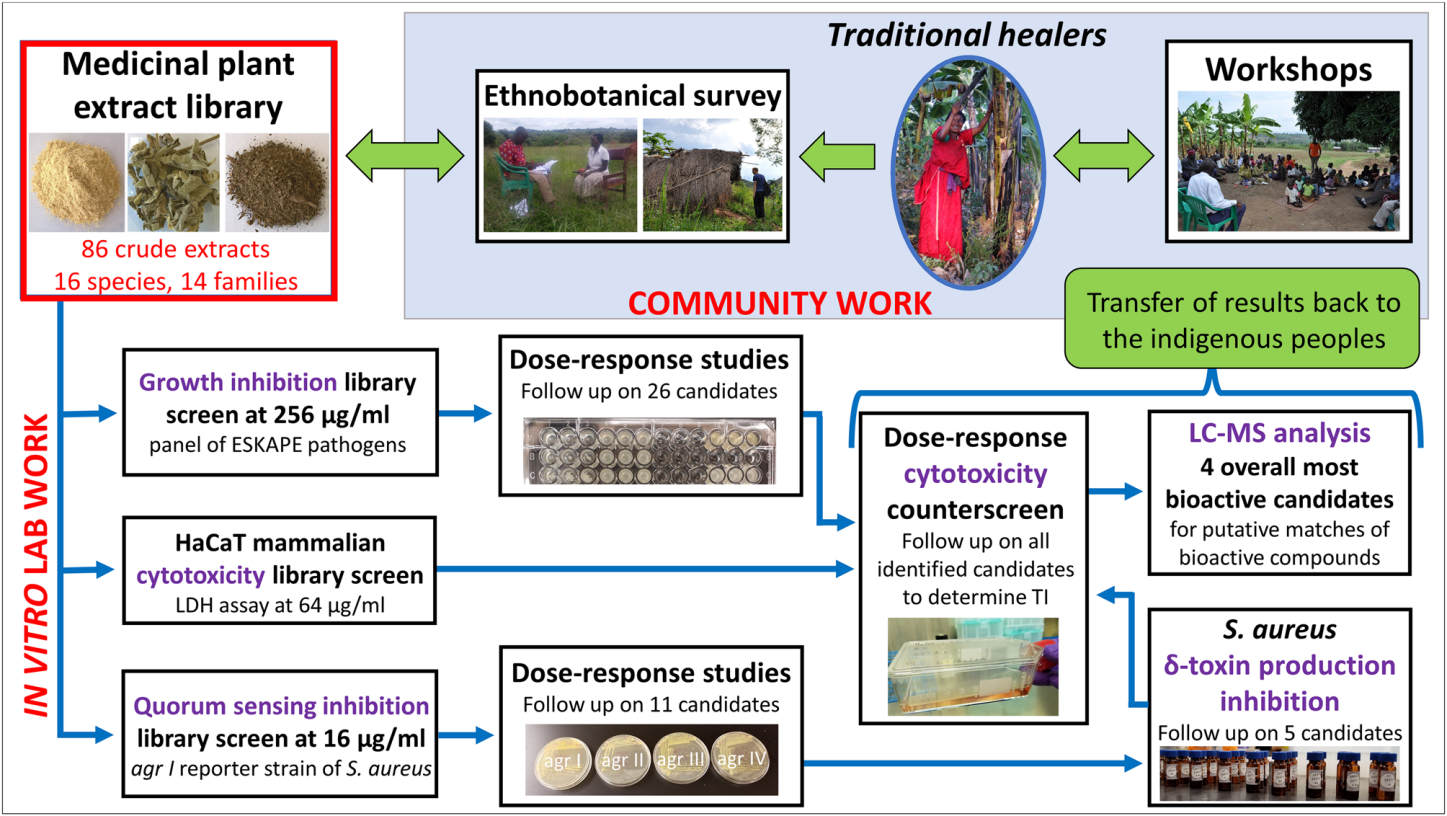
Research methodology for the study—16 plant species were identified in close collaboration with the traditional healers of the Greater Mpigi region based on the species’ traditional use in treatment of infections. After collecting specimens and producing a medicinal plant extract library, our in vitro study commenced, targeting bacterial virulence and growth of multidrug-resistant ESKAPE pathogens. After initial growth inhibition, quorum quenching and cytotoxicity library screenings, hits were followed up via dose–response studies, a δ-toxin production inhibition assay and chemical characterization. Results of this study will ultimately be transferred back to the traditional healers through field workshops.

We screened 86 plant extracts derived from these 16 medicinal species for antibacterial activity against a panel of multidrug resistant ESKAPE pathogens associated with the medical disorders stated in Fig. 1, and for antivirulence activity in *S. aureus* (Fig. 2). The extracts were produced from plant material collected in the Greater Mpigi region during fieldwork in 2015, 2016 and 2017. The overarching aims of the study were to contribute to drug discovery and pharmacological evaluation of traditional use. Specifically, the study objectives were to investigate the potential (1) growth inhibitory impact of the medicinal plants on a panel of ESKAPE pathogens; (2) quorum-quenching activity targeting the *agr* system of *S. aureus*; (3) mammalian cytotoxicity against the HaCaT keratinocyte cell line from adult human skin; (4) inhibition of δ-toxin production in *S. aureus*; and (5) to conduct a chemical characterization for putative natural product matches of the four most bioactive extracts.

## Results

### Extraction and information on plant species

Extractions were achieved by means of (a) maceration in either methanol, ethanol, ethyl acetate or diethyl ether; (b) Soxhlet extraction using *n*-hexane and successively methanol; and (c) aqueous decoction. These procedures yielded a total of 86 different plant crude extracts from 16 medicinal plant species. Details on the medicinal plants investigated, herbarium voucher specimen numbers, local plant names in Luganda, plant parts investigated, extract identification numbers (extract IDs) and solvents used for extraction are reported in Supplementary Table S1.

### Growth inhibition library screen and dose–response study against multi-resistant ESKAPE panel

Extracts were initially screened for growth inhibition of one clinical isolate of each ESKAPE pathogen at a concentration of 256 μg/mL*.* Extracts displaying an inhibition percentage above 40 for an individual strain were further investigated by dose–response experiments in order to obtain the IC_50_ and MIC (IC_90_) values. In this initial library screen, none of the extracts from *Ficus saussureana*, *Microgramma lycopodioides*, *Plectranthus hadiensis* and *Securidaca longipedunculata* displayed significant activity at this initial screening concentration and were therefore eliminated from further experiments. However, 26 of the 86 extracts were investigated further. In the second experimental stage, a total of 10 extracts from seven plant species inhibited the growth of *E. faecium* (EU-44)*.* While growth of *S. aureus* (UAMS-1) was significantly inhibited by 14 extracts from nine plant species at 256 μg/mL, only six extracts from three species were active against *K. pneumoniae* (CDC-004). Fifteen extracts from nine plant species were introduced to dose–response studies against *A. baumannii (*CDC-0033), and eight extracts from six plant species against *P. aeruginosa* (AH-71) respectively. Only the ethanolic and diethyl ether extracts from *Harungana madagascariensis* stem bark (etE011-18, dietE011) showed growth inhibition above 40% against *E. cloacae* (CDC-0032) at the initial screening concentration of 256 μg/mL. The individual plant extracts selected for the dose-response study and their results are shown in Table 1.

**Table 1:** Results of growth inhibition of selected ESKAPE pathogens by medicinal plant samples from the Greater Mpigi region in Uganda.

Scientific name	Extract ID	*Enterococcus faecium* EU-44		*Staphylococcus aureus* UAMS-1		*Klebsiella pneumoniae* CDC-004		*Acinetobacter baumannii* CDC-0033		*Pseudomonas aeruginosa* AH-71		*Enterobacter cloacae* CDC-0032	
		IC_50_	MIC	IC_50_	MIC	IC_50_	MIC	IC_50_	MIC	IC_50_	MIC	IC_50_	MIC
*Sesamum calycinum subsp. angustifolium*	eE004-18	–		256	> 256	–		–		–		–	
*Leucas calostachys*	eE005-18	–		–		–		> 256	> 256	–		–	
	hE005-18	128	256	256	> 256	> 256	> 256	–		256	> 256	–	
*Solanum aculeastrum*	hE006	256	256	32	128	–		–		–		–	
*Albizia coriaria*	etE007	–		–		–		> 256	> 256	32	> 256	–	
*Erythrina abyssinica*	etE008	64	> 256	32	64	–		> 256	> 256	–		–	
*Toddalia asiatica*	etE010	–		–		> 256	> 256	–		–		–	
	etE010a	–		–		> 256	> 256	–		–		–	
	eE010	–		–		> 256	> 256	–		–		–	
	dietE010	128	256	256	> 256	256	> 256	> 256	–	–		–	
*Harungana madagascariensis*	etE011-18	8	32	8	32	–		256	> 256	–		> 256	> 256
	eE011	128	128	–		–		–		–		–	
	dietE011	–		8	32	–		> 256	> 256	–		> 256	> 256
	dietE011-18	–		–		–		> 256	> 256	–		–	
	hE011-18	–		8	32	–		–		–		–	
*Morella kandtiana*	etE012	–		–		–		256	> 256	32	> 256	–	
	etE012a	–		–		–		256	> 256	32	> 256	–	
	etE012-18a	–		128	> 256	–		128	> 256	–		–	
	wE012-18	–		–		–		128	> 256	32	256	–	
*Cassine buchananii*	etE013	–		64	> 256	–		–		–		–	
*Warburgia ugandensis*	dietE014-18	128	> 256	32	64	–		–		–		–	
	eE014-18	128	> 256	–		–		–		–		–	
	hE014-18	–		64	64	–		> 256	> 256	–		–	
	etE014-18	256	256	128	128	256	> 256	128	–	64	> 256	–	
*Combretum molle*	etE015	–		–		–		32	> 256	16	128	–	
*Zanthoxylum chalybeum*	dietE017a	8	32	4	16	–		> 256	> 256	–	> 256	–	
Gentamicin		–		–		4	4	–		< 1	< 1	1,024	> 1,024
Meropenem		–		–		–		–		–		16	16
Vancomycin		–		4	4	–		–		–		–	
Ampicillin		–		–		–		–		> 256	> 256	–	
Tetracycline		–		–		–		2	4	–		–	
Chloramphenicol		4	32	–		–		–		–		–	

Only extracts that showed growth inhibition above 40% in the initial screen are listed. Crude extracts obtained during maceration were labeled according to their extraction solvent: (a) methanol (mEXXX); (b) ethanol (etEXXX); (c) ethyl acetate (eEXXX); (d) diethyl ether (dietEXXX), where ‘XXX’ stands for the sample number assigned to a given plant species. Crude extracts produced via Soxhlet extraction were labeled: (e) n-hexane (hEXXX); (f) methanol, successive extraction (smEXXX). In most cases, we recorded that the traditional healers prepare herbal drugs by boiling the plant material in water. Therefore, the original method of preparation was simulated by an aqueous decoction (wEXXX). Results are reported as the minimum concentration of extract that achieved 50% inhibition (IC50) and 90% inhibition (MIC) of growth as detected by optical density measures.

IC_50_ and MIC values are expressed as concentration (μg/mL). The maximum concentration at which extracts were tested was 256 μg/mL. Dashes indicate that a sample was not tested.

The diethyl ether extract of *Zanthoxylum chalybeum* stem bark (dietE017a) displayed the highest inhibitory activities in the study: *S. aureus* (IC_50_: 4 μg/mL; MIC: 16 μg/mL) and *E. faecium* (IC_50_: 8 μg/mL; MIC: 32 μg/mL). Ethanolic (etE011-18), diethyl ether (dietE011) and hexane extracts (hE011-18) of *H. madagascariensis* stem bark were the second most active extracts against growth of *S. aureus* (IC_50_: 8 μg/mL; MIC: 32 μg/mL) and the ethanolic stem bark extract displayed considerable antibiotic properties against *E. faecium*, resulting in the same IC_50_ (8 μg/mL) and MIC (32 μg/mL) values as *Z. chalybeum*. None of the extracts yielded an MIC at the concentration range tested (≤ 256 μg/mL) in the experiments with *K. pneumoniae*, *A. baumannii* and *E. cloacae*. Furthermore, 50% growth inhibition of *E. cloacae* was not achieved by the two *H. madagascariensis* stem bark extracts (etE011-18, dietE011) that were tested, meaning that none of the 86 extracts were active against the multidrug-resistant CDC-0032 strain. The ethanolic extract of *Combretum molle* stem bark (etE015) exhibited modest activity against *A. baumannii* (IC_50_: 32 μg/mL; MIC: > 256 μg/mL). Growth of *P. aeruginosa* was moderately inhibited by the ethanolic extract of *C. molle* stem bark (etE015; IC_50_: 16 μg/mL; MIC: 128 μg/mL) and *Morella kandtiana* roots (etE012-18a; IC_50_: 32 μg/mL; MIC: 256 μg/mL).

### Quorum sensing inhibition in *Staphylococcus aureus*

In *S. aureus,* a number of quorum-sensing component pathways are encoded by the accessory gene regulator (*agr*) system, which plays a key role in the species’ pathogenesis^14^. There are four allelic groups on the *agr* gene locus: *agr* I–IV^27^. The importance of the *agr* system to abscess formation has previously been confirmed by means of genetic and *agr*-inhibiting tools^28–32^.

During an initial screening, all 86 extracts were tested for inhibition of quorum sensing against the strain *S. aureus agr* I reporter strain AH-1677 at 16 μg/mL (sub-IC_50_ concentrations for growth were used to avoid potential growth inhibition effects). A total of 11 extracts from seven plant species revealed quorum-sensing inhibition activity above 40% and were selected for dose-response experiments with four reporter strains of *S. aureus agr* subtypes (*agr* I: AH-1677, *agr* II: AH-430, *agr* III: AH-1747, *agr* IV: AH-1872). These plant species, which were significantly active in the initial screen, were *Sesamum calycinum subsp. angustifolium* (both hexane leave extracts; hE004 and hE004-18), *Leucas calostachys* (hexane leave extract; hE005), *Solanum aculeastrum* (hexane root extract; hE006, and ethyl acetate root extract; eE006), *Z. chalybeum* (ethyl acetate stem bark extract; eE009)*, M. kandtiana* (both diethyl ether root extracts; dietE012 and dietE012-18)*, Warburgia ugandensis* (diethyl ether stem bark extract; dietE014) and *P. hadiensis* (hexane leave extract; hE016, and diethyl ether leave extract; dietE016) (Table 2). None of the extracts from *S. longipedunculata*, *M. lycopodioides, F. saussureana*, *Albizia coriaria, Erythrina abyssinica, T. asiatica, H. madagascariensis* and *C. molle* inhibited quorum sensing above 40% at 16 μg/mL.

**Table 2:** Results of quorum-sensing inhibition plant extract library screen on *S. aureus agr* I reporter strain at 16 μg/mL.

Plant species	Extract ID	%*I * ≥ 40	Plant species	Extract ID	%*I * ≥ 40	Plant species	Extract ID	%*I * ≥ 40	Plant species	Extract ID	%*I * ≥ 40
*Securidaca longipedunculata*	eE001	**–**	*Leucas calostachys*	eE005	**–**	*Toddalia asiatica*	etE010	**–**	*Cassine buchananii*	etE013	**–**
	smE001	**–**		eE005-18	**–**		etE010a	**–**		etE013a	**–**
	wE001	**–**		smE005	**–**		eE010	**–**		eE013	**–**
	mE001	**–**		smE005-18	**–**		dietE010	**–**	*Warburgia ugandensis*	dietE014	+
	hE001	**–**		wE005	**–**	*Harungana madagascariensis*	etE011	**–**		dietE014-18	**–**
*Microgramma lycopodioides*	hE002	**–**		mE005-18	**–**		etE011a	**–**		eE014-18	**–**
	mE002	**–**		hE005	+		etE011-18	**–**		wE014-18	**–**
	wE002	**–**		hE005-18	**–**		eE011	**–**		hE014-18	**–**
	smE002	**–**	*Solanum aculeastrum*	eE006	+		eE011-18	**–**		smE014-18	**–**
	eE002	**–**		hE006	+		dietE011	**–**		etE014a	**–**
*Ficus saussureana*	smE003	**–**		wE006	**–**		dietE011-18	**–**		etE014-18	**–**
	wE003	**–**		smE006	**–**		wE011-18	**–**	*Combretum molle*	etE015	**–**
	eE003	**–**	*Albizia coriaria*	etE007	**–**		hE011-18	**–**		eE015	**–**
	mE003	**–**		eE007	**–**		smE011-18	**–**	*Plectranthus hadiensis*	hE016	+
	hE003	**–**	*Erythrina abyssinica*	etE008	**–**	*Morella kandtiana*	etE012	**–**		dietE016	+
*Sesamum calycinum subsp. angustifolium*	smE004	**–**		eE008	**–**		etE012a	**–**			
	smE004-18	**–**	*Zanthoxylum chalybeum*	etE009	**–**		etE012-18a	**–**			
	mE004	**–**		eE009	+		etE012-18b	**–**			
	hE004	+		etE017	**–**		eE012-18	**–**			
	hE004-18	+		etE017a	**–**		wE012-18	**–**			
	eE004	**–**		dietE017	**–**		dietE012	+			
	eE004-18	**–**		dietE017a	**–**		dietE012-18	+			
	wE004	**–**									

–, Quorum-sensing inhibition below 40 percent;  + , quorum-sensing inhibition above 40 percent.

### Ugandan medicinal plant species exhibit dose-dependent quorum-sensing inhibition in vitro

**Figure 3:**
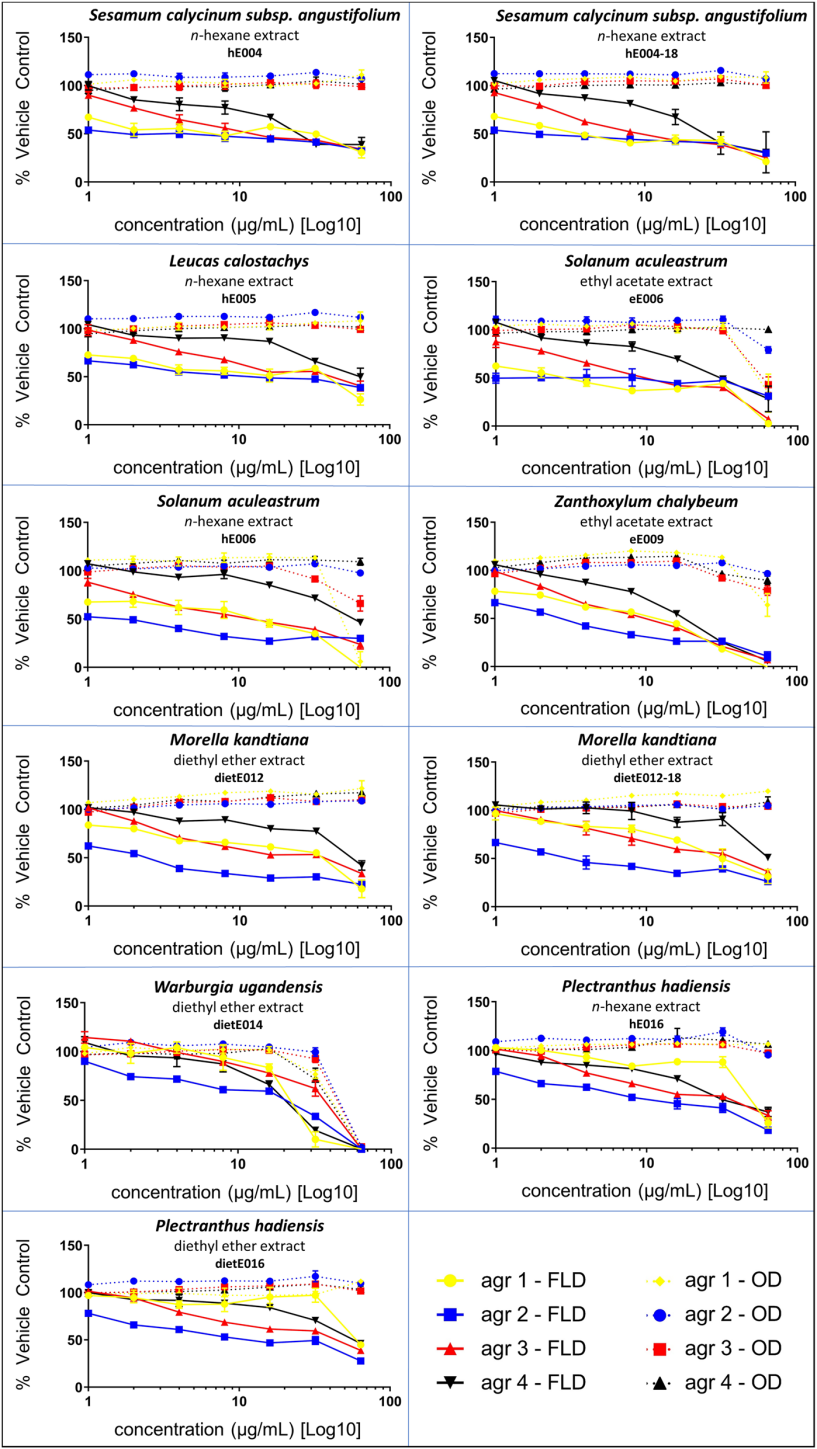
Results of the quorum-sensing inhibition in vitro dose-response studies: Data shown as serial dilution and percent *agr* activity or growth of the vehicle control (DMSO) at 22 h; FLD: fluorescence detector (measuring quorum sensing activity), represented by solid lines; OD: optical density at 600 nm (measuring bacterial growth), represented by dashed lines.

The transcription of each of the four known *agr* allelic groups was inhibited by all of the selected 11 crude extracts from seven plant species. Strains were additionally monitored for potential growth inhibition by optical density (600 nm). Dose-response curves, indicating the percent growth inhibition and quorum sensing inhibition (QSI) activity of the vehicle control (dimethyl sulfoxide [DMSO]), were calculated to evaluate the antivirulence activity (Fig. 3). *Agr* subtype-specific IC_50_ values are reported in Table 3. The two hexane extracts of *S. calycinum subsp. angustifolium* leaves, hE004 and hE004-18, were identified as the most active quorum-sensing inhibitors. The IC_50_ against *agr* I–IV were 2, 2, 16 and 32 μg/mL (hE004), 4, 2, 16 and 32 μg/mL (hE004-18) respectively. Another plant extract highly active in tackling bacterial virulence was the ethyl acetate extract of *S. aculeastrum* roots (eE006), which scored *agr* subtype-dependent IC_50_ values of 4, 1, 16 and 64 μg/mL. Two more promising quorum-sensing inhibitors were the hexane extract of *S. aculeastrum* roots (hE006, *agr* I–III IC_50_: 12, 2 and 16 μg/mL), which only displayed moderate activity against *agr* IV (IC_50_: 64 μg/mL), and the hexane extract of *L. calostachys* leaves (hE005, *agr* I–II: 4 μg/mL), which was moderately active against *agr* III and IV (IC_50_: 64 μg/mL). Extracts of *W. ugandensis* and *Z. chalybeum* stem bark showed low IC_50_ values ranging from 8–32 μg/mL, but were eliminated from the anti-*agr* assessment due to their strong growth inhibitory activity on our reporter strains. No MIC values were detected (either > 64 μg/mL or growth inhibition), except for the hexane extract of *S. calycinum subsp. angustifolium* leaves (hE004-18, MIC: 64 μg/mL).

**Table 3:** Results of the quorum-sensing inhibition in vitro dose-response studies: IC_50_ and MIC values.

Plant species	Extract ID	*agr 1* AH 1677		*agr 2* AH 430		*agr 3* AH 1747		*agr 4* AH 1872	
		IC_50_	MIC	IC_50_	MIC	IC_50_	MIC	IC_50_	MIC
*Sesamum calycinum subsp. angustifolium*	hE004	**2**	** > 64**	**2**	** > 64**	**16**	** > 64**	**32**	** > 64**
	hE004-18	**4**	**64**	**2**	** > 64**	**16**	** > 64**	**32**	** > 64**
*Leucas calostachys*	hE005	**4**	** > 64**	**4**	** > 64**	**64**	** > 64**	**64**	** > 64**
*Solanum aculeastrum*	eE006	**4**	> 32 GI	**1**	> 32 GI	**16**	> 32 GI	**64**	** > 64**
	hE006	**16**	> 32 GI	**2**	** > 64**	**16**	> 32 GI	**64**	** > 64**
*Zanthoxylum chalybeum*	eE009	**16**	> 32 GI	**8**	** > 64**	**16**	> 32 GI	**16**	> 16 GI
*Morella kandtiana*	dietE012	**64**	** > 64**	**4**	** > 64**	**16**	** > 64**	**64**	** > 64**
	dietE012-18	**64**	** > 64**	**4**	** > 64**	**64**	** > 64**	**64**	** > 64**
*Warburgia ugandensis*	dietE014	**32**	> 32 GI	**32**	> 32 GI	> 32 GI	> 32 GI	> 16 GI	> 16 GI
*Plectranthus hadiensis*	hE016	**64**	** > 64**	**16**	** > 64**	**64**	** > 64**	**64**	** > 64**
	dietE016	**64**	** > 64**	**16**	** > 64**	**64**	** > 64**	**64**	** > 64**
224CF2c (positive control)		**16**	> 32 GI	**16**	> 32 GI	**16**	> 32 GI	**32**	> 32 GI

The calculated IC_50_ and MIC values of plant extracts, represented in μg/mL, are displayed. The most active extracts were selected for confirmation of antivirulence activity via a δ-toxin production and quantification assay; > 16/32 GI describes undetectable IC_50_ and MIC values due to growth inhibition at 16 or 32 μg/mL.

GI, growth inhibition.

### δ-Toxin production and quantification assay

**Figure 4:**
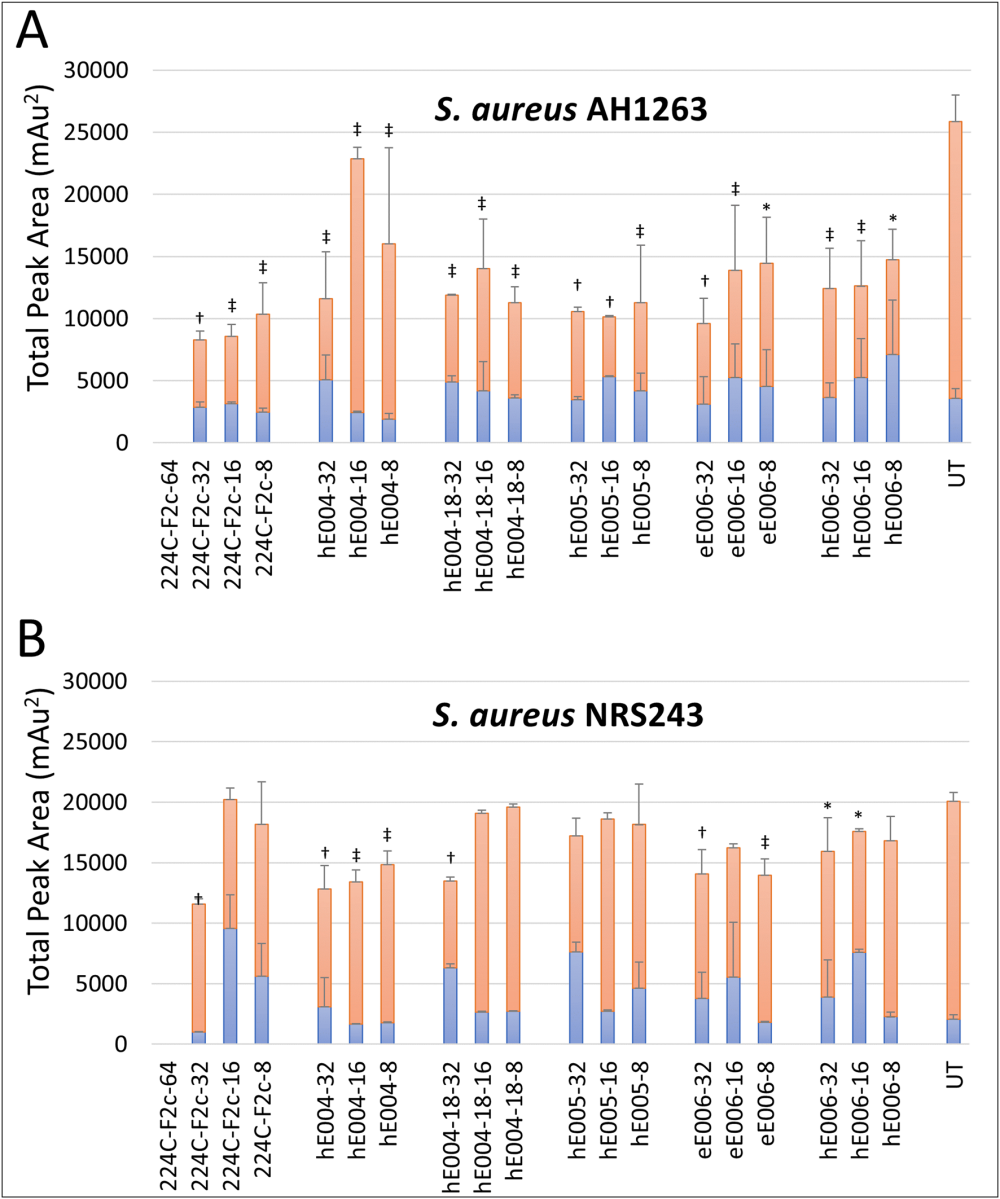
Five extracts from three Ugandan medicinal plant species exhibited strong δ-toxin production inhibition activity against *S. aureus* AH1262 (**A**) and moderate activity against *S. aureus* NRS243 (**B**); extracts were tested at 32, 16 and 8 μg/mL (sub-growth inhibition concentrations) and compared to the untreated control (UT). The positive control 224CF2c was additionally tested at 64 μg/mL. All samples were normalized for growth (OD_600nm_) during supernatant harvest. Results are reported as the total peak area and peaks are identified as deformylated (blue) and formylated (red) δ-toxin peak areas. Statistical significance is denoted as *P value < 0.05, ^‡^*P* value < 0.01, *†P* value < 0.001.

The phenol-soluble modulin peptide δ-toxin (also known as δ-hemolysin) is responsible for various pathophysiological effects caused by *S. aureus* as it seeks to evade host defense mechanisms^33–36^. These effects include cytolysis of red and white blood cells, followed by cell death, as well as triggering of inflammatory responses^33,36^. Extracts hE004, hE004-18, hE005, eE006, hE006, which displayed strong quorum sensing inhibitory activity (Table 3), were selected for further confirmation of antivirulence effects on the translational products of *agr* in *S. aureus*. These in vitro experiments aimed to measure δ-toxin levels during extract treatment at sub-growth inhibitory concentrations through examination of the bacterial supernatant using hydrophobic interaction chromatography (HIC)^37^. The experiments were conducted with two high-toxin-producing strains of *S. aureus*: AH1263 and NRS243. All tested extracts were effective in significantly reducing δ-toxin in AH1263, confirming their antivirulence activity. The hexane extracts of *S. calycinum subsp. angustifolium* leaves (hE004, hE004-18) and the ethyl acetate extract of *S. aculeastrum* roots (eE006) displayed the highest inhibition activity against NRS243. Extracts hE005 and hE006 showed moderate activity against NRS243 (Fig. 4).

### Medicinal plants from the Greater Mpigi region exhibit low toxicity to human keratinocytes

In an effort to assess the cytotoxicity of the plant extracts, all 86 extracts were screened in a human keratinocyte toxicity assay at 64 μg/mL, using HaCaT cells. In this library screen, only one of the 86 extracts from 16 plant species exhibited a cytotoxicity above 50%. The only extract displaying cytotoxic activity in the initial screen was the methanolic extract of *S. aculeastrum* roots smE006 (I%: 51.8 ± 1.5). Results of the cytotoxicity library screen are shown in Supplementary Table S3. Subsequently, dose-response experiments to assess cytotoxicity were conducted on extract smE006, the 26 active hits from the growth inhibition library screen (see Table 1) and the five most active quorum-sensing inhibitors that were introduced to the δ-toxin production inhibition assay (see Figs. 3 and 4, Table 3). Results of this counterscreen are shown in Table 4, along with the calculated therapeutic indices for growth inhibition (TI_growth inhibition_) for individual strains tested and quorum-sensing inhibition (TI_quorum quenching_) for each reporter gene targeted. The therapeutic index is used as an important parameter in drug discovery to assess an appropriately balanced safety-efficacy profile for a given indication, as it enables for characterization and optimization of efficacy and safety of drug candidates^38^. The majority of extracts tested in our dose-response study displayed no toxicity to the HaCaT cells (20 extracts, 64.5%). However, some extracts did show low toxicity with IC_50_ values ranging from 512 to 256 μg/mL: (1) the ethyl acetate extract of *S. calycinum subsp. angustifolium* leaves (eE004-18); (2) three extracts of *L. calostachys* leaves (eE005-18, hE005, hE005-18); (3) two extracts of *S. aculeastrum* roots (hE006, smE006); (4) the diethyl ether extract of *Toddalia asiatica* leaves/stem bark (dietE010); and (5) four extracts of *H. madagascariensis* stem bark (etE011-18, dietE011, dietE011-18, hE011-18). As expected, extract smE006 remained the most cytotoxic sample in the extract library, displaying an IC_50_ of 64 μg/mL.

**Table 4:** The 30 most active Ugandan plant extracts are either non-toxic or show low toxicity to human HaCaT cells (Table showing results of cytotoxicity dose–response experiments and calculated therapeutic indices).

Plant species	Extract ID	Cytotoxicity	TI_growth inhibition_	TI_quorum quenching_			
		IC_50_		*agr* I	*agr* II	*agr* III	*agr* IV
*Sesamum calycinum subsp. angustifolium*	hE004	> 512	–	> 256	> 256	> 32	> 16
	hE004-18	> 512	–	> 128	> 256	> 32	> 16
	eE004-18	512	2*	–	–	–	–
*Leucas calostachys*	eE005-18	256	< 1°	–	–	–	–
	hE005	256	–	64	64	4	4
	hE005-18	512	4^†^; 2*; < 2^§^; 2^ǂ^	–	–	–	–
*Solanum aculeastrum*	eE006	> 512	–	> 128	> 512	> 32	> 8
	hE006	512	2^†^; 16*	32	256	32	8
	smE006	64	–	–	–	–	–
*Albizia coriaria*	etE007	> 512	--°; > 16^ǂ^	–	–	–	–
*Erythrina abyssinica*	etE008	> 512	> 8^†^; > 16*; --°	–	–	–	–
*Toddalia asiatica*	etE010	> 512	--^§^	–	–	–	–
	etE010a	> 512	--^§^	–	–	–	–
	eE010	> 512	--^§^	–	–	–	–
	dietE010	256	2^†^; 1*; 1^§^; < 1°	–	–	–	–
*Harungana madagascariensis*	etE011-18	256	32^†^; 32*; 1°; < 1^**±**^	–	–	–	–
	eE011	> 512	> 4^†^;	–	–	–	–
	dietE011	256	32*; < 1°; < 1^**±**^	–	–	–	–
	dietE011-18	256	< 1°	–	–	–	–
	hE011-18	256	32*	–	–	–	–
*Morella kandtiana*	etE012	> 512	> 2°; > 16^ǂ^	–	–	–	–
	etE012a	> 512	> 2°; > 16^ǂ^	–	–	–	–
	etE012-18a	> 512	> 4*; > 4°	–	–	–	–
	wE012-18	> 512	> 4°; > 16^ǂ^	–	–	–	–
*Cassine buchananii*	etE013	> 512	> 8*	–	–	–	–
*Warburgia ugandensis*	dietE014-18	> 512	> 4^†^; > 16*	–	–	–	–
	eE014-18	> 512	> 4^†^	–	–	–	–
	hE014-18	> 512	> 8*; --°	–	–	–	–
	etE014-18	> 512	> 2^†^; > 4*; > 2^§^; > 4°; > 8^ǂ^	–	–	–	–
*Combretum molle*	etE015	> 512	> 16°; > 32^ǂ^	–	–	–	–
*Zanthoxylum chalybeum*	dietE017a	> 512	> 64^†^; > 128*; --°; --^ǂ^	–	–	–	–

IC_50_ values are given in µg/mL.

^†^*E. faecium* EU-44; *S. aureus UAMS-1; ^§^*K. pneumoniae* CDC-004; °*A. baumannii* CDC-033; ^ǂ^*P. aeruginosa* AH-71; ^±^*E. cloacae* CDC-0032; --, cannot be calculated; –, not tested.

### LC–MS analysis of plant extracts for putative matches

**Figure 5:**
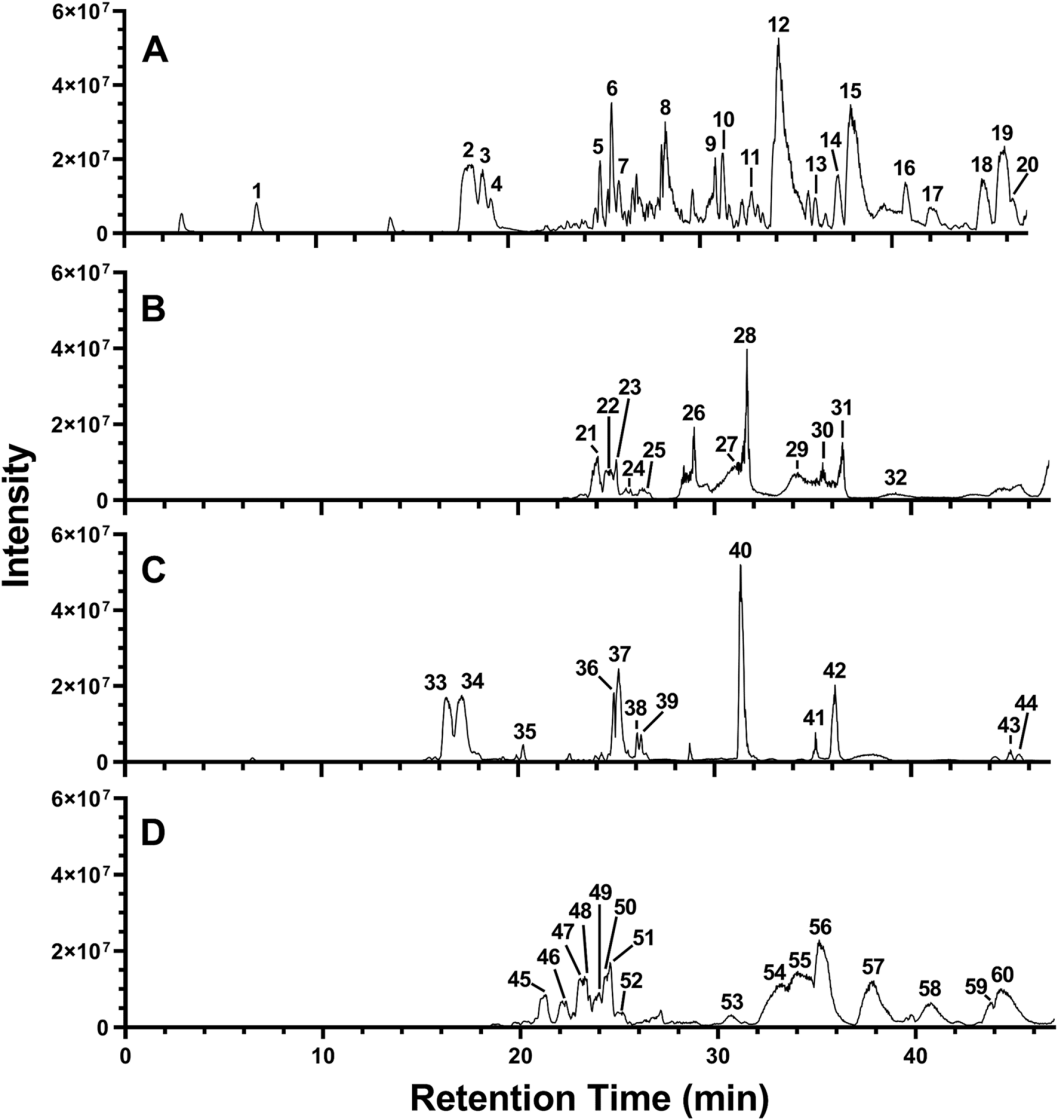
The ESI negative mode base peak LC-FTMS chromatogram for (**A**) etE011-18, *H. madagascariensis*, (**B**) hE004-18, *S. calycinum subsp. angustifolium*, (**C**) eE006, *S. aculeastrum*, and (**D**) dietE017a, *Z. chalybeum*.

The two best performing extracts of the growth inhibition experiments (dietE017a and etE011-18), as well as of the quorum-sensing and δ-toxin production assays (hE004-18 and eE006) were further investigated by chemical characterization via liquid chromatography–mass spectrometry (LC–MS) analysis and searched for putative matches. The base peak negative mode electrospray ionization (ESI) LC–MS chromatograms for the four extracts are shown in Fig. 5. A total of 60 peaks were identified and screened for putative matches (Fig. 5 and Table 5). This resulted in 10 peaks having putative matches for etE011-018, 9 peaks for hE004-18, 9 peaks for eE006, and 2 peaks for dietE017a. Most of the ions yielded several putative matches which are isomers of the experimentally determined empirical formula. The only putative match for dietE017a, peak **54** and **55**, was cyclozanthoxylane A, which has a mass difference of over 13 ppm from the experimentally determined mass. While this is a low probability match, it was the only putative match for the *Z. chalybeum* sample from 9,463 published compounds in the genus. Chemical structures for the putative matches from the four extracts are provided in Supplementary Figures S3, S4, S5 and S6.

**Table 5:** LC–MS data and putative matches for extracts etE011-18, hE004-18, eE006, and dietE017a.

Peak no.	RT (min)	% Area	m/z*	MS–MS	Empirical formula	Putative match (CAS no.)
**etE011-18, *Harugana madagascariensis***						
1	6.9	1.6	**465.1050**, 931.2171	345.1, 375.1	C_21_H_21_O_12_ (2.4)	no matches
2	18.0	8.4	**589.1005,** 1,179.2081	463.1	C_30_H_21_O_13_ (2.9)	no matches
3	18.7	3.6	573.1053, **589.1004,** 1,147.2178	463.1	C_30_H_21_O_13_ (2.9)	no matches
4	19.1	1.5	447.0734, 573.1050, **589.1006**	463.1	C_30_H_21_O_13_ (3.0)	no matches
5	24.8	1.7	**424.1896,** 493.2603	381.2	C_25_H_28_O_6_ (1.1)	calycinigin A (1384180-74-8)
6	25.4	3.5	**475.2498,** 493.2606	406.2	C_30_ H_35_ O_5_ (1.8)	bazouanthrone (942983-94-0), Kenganthranol B (879208-71-6)
7	25.8	1.3	**475.2498,** 895.5402	406.2	C_30_ H_35_ O_5_ (1.8)	see peak 7
8	28.2	5.9	322.1213, **391.1919**	322.1	C_25_H_27_O_4_ (1.1)	no matches
9	30.8	1.0	**406.1793,** 455.3537	363.2	C_25_ H_26_ O_5_ (1.8)	mammeisin (18483-64-2), 3-geranylemodin (87605-71-8), 2-geranylemodin (97399-77-4), kengaquinone (879208-69-2)
10	31.2	1.8	491.2447	423.2, 473.3	C_30_H_35_O_6_ (1.6)	no matches
11	32.7	1.3	**475.2498,** 797.3711	292.1, 347.2	C_30_ H_35_ O_5_ (1.7)	see peak 7
12	34.1	22.3	390.1841, **459.2547**	390.2	C_30_H_35_O_4_ (1.4)	harunganin (3736-60-5), harungin anthrone (59204-72-70), ferruginin B (73210-80-7), ferruginin A (73210-81-8), harunganol B (84393-25-9)
13	36.0	1.0	781.3761	322.1, 712.2	C_50_H_53_O_8_ (2.0)	bianthrone A_1_ (97399-74-1**)**
14	37.2	1.7	**475.2501,** 489.2292, 933.4983	457.3	C_30_H_35_O_5_ (2.2)	see peak 7
15	37.9	13.4	390.1841, **459.2548**	390.2	C_30_H_35_O_4_ (1.5)	see peak 12
16	40.7	2.5	**601.3555,** 917.5029	409.2, 465.4	C_38_H_49_O_6_ (3.3)	xanthochymol (52617-32-0), cambogin (71117-97-0), garcinol (78824-30-3), guttiferone F (219538-86-0), coccinone F (1141870-97-4), coccinone G (1141870-99-6), coccinone H (1141871-01-3), coccinone A (1141871-31-9)
17	42.0	1.9	865.4342	407.2, 796.3	C_55_H_61_O_9_ (2.5)	no matches
18	44.8	2.7	849.4391	322.1, 780.3	C_55_H_61_O_8_ (2.1)	no matches
19	45.9	7.7	527.3179, **865.4345**	407.3, 796.2	C_55_H_61_O_9_ (2.7)	no matches
20	46.3	1.8	527.3179, 865.4345, **949.4933**	422.2, 880.3	C_53_H_73_O_15_ (-2.5)	no matches
**hE004-18,** ***Sesamum calycinum*** **subsp.** ***angustifolium***						
21	24.1	12.4	293.2123, **309.2073**, 609.4149, 844.6181	209.1, 291.2	C_18_H_29_O_4_ (0.6)	tetrahydrotrisporic acid C (35996-92-0)
22	24.6	12.7	295.2283, 564.4143, **860.6514**	ND	C_55_H_88_O_7_ (-2.5)	no matches
23	25.0	5.3	295.2281	171.0, 195.1, 277.2	C_18_H_31_O_3_ (0.68)	vernolic acid (503-07-1)
24	25.5	1.8	293.2124, 471.3490, **564.4149**	270.3, 547.2	C_37_H_56_O_4_ (5.2)	no matches
25	26.3	3.9	295.2281	171.1, 195.1, 251.2, 277.2	C_18_H_31_O_3_ (0.68)	see peak 23
26	29.0	9.0	**277.2175,** 933.4965	233.2, 259.2	C_18_H_29_O_2_ (0.6)	alpha-linolenic acid (463-40-1), eleostearic acid (506-23-0), gamma-linolenic acid (506-26-3), trichosanic acid (544-72-9), beta-eleostearic acid (544-73-0), 9,​12,​15-​octadecatrienoic acid (1955-33-5), 5,​9,​12-​octadecatrienoic acid (13237-97-3), elaeostearic acid (13296-76-9), linolenelaidic acid (28290-79-1)
27	31.5	5.8	279.2332, **455.3539,** 933.4983	407.4	C_30_H_47_O_3_ (1.8)	betulinic acid (472-15-1), oleanic acid (508-02-1), boswellic acid (631-69-6)
28	31.7	16.7	279.2331, **455.3536,** 933.4961	407.4	C_30_H_47_O_3_ (1.1)	see peak 27
29	34.2	10.9	933.4965, **949.4937**	ND	C_53_H_73_O_15_ (-1.9)	no matches
30	35.5	6.7	**255.2330,** 281.2488	237.2, 255.3	C_16_H_31_O_2_ (0.1)	palmitic acid (57-10-3), ethyl myristate (124-06-1), methyl pentadecanoate (7132-64-1)
31	36.5	10.9	**281.2487,** 865.4341	263.3, 281.3	C_18_H_33_O_2_ (2.4)	elaidic acid (112-79-8), oleic acid (112-80-1), 11Z-octadecenoic acid (506-17-2), (6Z)-6-octadecenoic acid (593-39-5), (11E)-11-octadecenoic acid (693-72-1), methyl 9,10-methylenehexadecanoate (10152-61-1), (7Z)-7-octadecenoic acid (13126-31-3), ethyl 9-hexadecenoate (54546-22-4)
32	39.2	3.8	**455.3544,** 865.4335	393.3, 407.4, 409.4, 437.4	C_30_H_47_O_3_ (4.1)	see peak 27
**eE006,** ***Solanum aculeastrum***						
33	16.3	14.5	766.4394, **912.4975**	866.4	C_43_H_76_O_20_ (4.4)	no matches
34	17.1	16.3	**720.4350,** 766.4398	246.8, 574.1	C_39_H_62_NO_11_ (2.8)	γ_2_-Solamarine (11034-34-7), γ_1_-solamarine (15299-06-6), β2-solamargine (32449-98-2), β_2_-solanine (61877-94-9), β_1_-solasonine (73069-18-8), β_1_-solamargine (73069-20-2), β-d-glucopyranoside derivative of solanidane (81920-14-1), β-d-tomatid-​5-​en-​3β-​ol 4-​o-​α-​l-​rhamnopyranosyl-​glucopyranoside (906342-97-0)
35	20.3	1.6	**299.0563,** 599.1220	284	C_16_H_11_O_6_ (0.7)	3′-Methoxyapigenin (491-71-4), diosmetin (520-34-3), 7-methylkaempferol (569-92-6), 6-methoxyapigenin (1447-88-7), 3-methylkaempferol (1592-70-7), 8-hydroxyacacetin (51876-19-8), 5,8,4′-trihydroxy-7-methoxyflavone (56595-23-4)
36	24.9	4.8	295.228	171.1, 195.1, 277.2	C_18_H_31_O_3_ (0.4)	trans-3-​oxo-​2-​pentyl-​cyclopentaneoctanoic acid (91403-58-6), (1R,​5R)​-​rel-2-​oxo-​5-​pentyl-cyclopentaneoctanoic acid (282091-22-9), vernoleic acid (503-07-1), coronaric acid (16833-56-0), α-artemisolic acid (18104-45-5), (±)-α-dimorphecolic acid (98524-19-7), (Z,E)-9-hydroxy-10,12-octadecadienoic acid (109281-79-0)
37	25.1	14.0	293.2123, **311.2230**	293.2	C_18_H_31_O_4_ (0.8)	9-​Octadecenedioic acid (4494-16-0), (9Z)​-13-​hydroxy-​12-​oxo-9-​octadecenoic acid (5502-89-6), (E,Z)-9-hydroperoxy-10,12-octadecadienoic acid (5502-91-0), 9-​hydroperoxy-10,​12-​octadecadienoic acid (7324-20-1), 13-hydroperoxylinoleic acid (7324-21-2), 9,​11-​13-​(9Z,​11E)​-hydroperoxy-​octadecadienoic acid (23017-93-8), (11E)​-13-​hydroxy-​10-​oxo-11-​octadecenoic acid (28979-44-4), (9S,​10E,​12Z)​-9-​hydroperoxy-10,​12-​octadecadienoic acid (29774-12-7), (9Z,​11E,​13S)​-13-​hydroperoxy-9,​11-​octadecadienoic acid (33964-75-9), 9-D-hydroperoxy-10,12-octadecadienoic acid (39692-45-0), (10E,​12E)​-9-​hydroperoxy-​10,​12-​octadecadienoic acid (63121-49-3), (9R,​12Z)​-9-​hydroxy-​10-​oxo-​12-​octadecenoic acid (70144-92-2), (θS,​2S,​3S)​-3-​(1Z)​-​1-​hepten-​1-​yl-​θ-​hydroxy-2-​oxiranenonanoic acid (282091-26-3), 3-​[(1R,​2Z)​-​1-​hydroxy-​2-​octenyl]​-​(2S,​3R)​-oxiraneoctanoic acid (166735-97-3)
38	26.1	1.7	295.2281, **313.2388**	295.2	C_18_H_33_O_4_ (1.2)	1,​10-​dibutyl decanedioic acid ester (109-43-3), 1,​6-​dihexyl-hexanedioic acid ester (110-33-8), 16-​hydroxy-​9-​oxo-octadecanoic acid (132796-50-0), 9-​hydroxy-​16-​oxo-octadecanoic acid (132828-40-1)
39	26.3	2.1	295.2282, **313.2388**	295.2	C_18_H_33_O_4_ (1.3)	see peak 38
40	31.3	26.1	279.233	261.2	C_18_H_31_O_2_ (0.1)	stereo isomers of 9,​12-octadecadienoic acid (2197-37-7)
41	35.1	2.1	255.2331, **458.2477,** 915.4877	403.3, 415.2	C_30_H_34_O_4_ (3.6)	no matches
42	36.1	10.2	281.2487	263.3, 281.3	C_18_H_33_O_2_ (0.4)	stereo isomers of 9-octadecenoic acid (112-79-8)
43	45.1	1.3	283.2645, **933.4976**	846.3	C_46_H_77_O_19_ (-9.5)	β-d-glucopyranoside, (3β, 5α, 6α,​22α,​25*S*)​-​26-​(β-d-​glucopyranosyloxy)​-​3-​hydroxy-​22-​methoxyfurostan-​6-​yl 3-​*O*-​(6-​deoxy-​α-​L-​mannopyranosyl) (1418008-88-4)
44	45.5	1.1	283.2647, **607.4019**	589.3	C_38_H_55_O_6_ (2.5)	no matches
**dietE017a,** ***Zanthoxylum chalybeum***						
45	21.3	3.8	**465.2293,** 931.4689	406.3, 421.3	C_28_H_33_O_6_ (2.3)	no matches
46	22.1	3.3	421.2399, **451.2500,** 903.5092	407.3, 436.3	C_28_H_35_O_5_ (2.2)	no matches
47	23.0	3.1	435.2552, 451.2507, **453.2667,** 871.5184	ND	C_28_H_37_O_5_ (4.5)	no matches
48	23.2	2.1	435.2552, **465.2665,** 871.5193, 901.5299	ND	C_29_H_37_O_5_ (4.0)	no matches
49	24.0	2.5	437.2709, **453.2655**	315.3, 425.3, 435.3	C_28_H_37_O_5_ (2.0)	no matches
50	24.3	2.2	437.2709, **453.2655**	315.3, 425.3, 435.3	C_28_H_37_O_5_ (2.0)	no matches
51	24.5	3.3	**449.2344,** 899.4781	406.3, 434.2	C_28_H_33_O_5_ (2.4)	no matches
52	25.2	1.7	451.2499	436.3	C_28_H_35_O_5_ (2.0)	no matches
53	30.7	2.0	933.4972, **949.4942**	ND	C_53_H_73_O_15_ (-1.4)	no matches
54	33.2	9.7	390.1845, **459.2550**	390.2	C_23_H_33_N_5_O_5_ (13.6)	cyclozanthoxylane A (1384258-42-7)
55	34.0	14.1	**459.2551,** 933.4975	403.2	C_23_H_33_N_5_O_5_ (13.8)	see peak 54
56	35.1	16.0	458.2473, **527.3181,** 933.4977	458.2	C_35_H_43_O_4_ (2.6)	no matches
57	37.9	11.1	**849.4393,** 933.4981	390.2, 780.2	C_55_H_61_O_8_ (2.5)	no matches
58	40.8	5.7	933.4972	407.3, 864.3	C_60_H_69_O_9_ (2.6)	no matches
59	43.8	2.2	**503.2812,** 933.4977	459.2	C_32_H_39_O_5_ (1.8)	no matches
60	44.3	10.4	933.4974	407.3, 864.3	C_60_H_69_O_9_ (2.9)	no matches

ND, not detected.

*When multiple base ions were detected, the *m/z* in **bold** fond indicates the ion used to predict the empirical formula and which underwent MS^2^ fragmentation.

## Discussion

The study provides scientific evidence for the therapeutic use of medicinal plants in the Ugandan Greater Mpigi region. Traditional use in treatment of infections and wounds was successfully validated in 13 out of 16 medicinal plant species investigated using in vitro studies. Extracts of species displaying no pharmacological activity in these experiments were *S. longipedunculata*, *M. lycopodioides* and *F. saussureana*. On the contrary, different extracts of *S. calycinum subsp. angustifolium*, *L. calostachys*, *S. aculeastrum*, *M. kandtiana*, *W. ugandensis* and *Z. chalybeum* simultaneously displayed both growth inhibition and quorum-sensing inhibition effects on the strains investigated. Extracts from the same species distinguished themselves in terms of polarity of extraction solvent used (“pre-fractionation”). Except for the hexane extract of *S. aculeastrum* roots hE006, there was no extract that was simultaneously active in inhibiting bacterial growth and quorum quenching, which highlights the need for bioassay-guided fractionation and isolation of active compounds from these species in the future.

In general, extracts produced as aqueous decoction, which is consistent with the majority of the traditional preparations in the Greater Mpigi region^24^, failed to display bioactive effects in our in vitro models. The exception was one aqueous extract of *M. kandtiana* roots, which exhibited low inhibitory effects on the growth of *A. baumannii* CDC-0033 (IC_50_: 128 μg/mL; MIC: > 256 μg/mL) and moderate effects on *P. aeruginosa* AH-71 (IC_50_: 32 μg/mL; MIC: 256 μg/mL). One possible factor that could contribute to this phenomenon is the fact that extracts are standardized in the lab (filtered before solvent evaporation), unlike during traditional treatment where solids are swallowed along with the infused water. In this way, apolar pharmacologically active secondary plant metabolites bound to the solids remain in the decoction and could potentially yield a pharmacological effect in the patient.

Extract dietE017a, a diethyl ether extract of *Z. chalybeum* stem bark, displayed the highest growth inhibitory activity of all extracts against growth of *S. aureus* (IC_50_: 4 μg/mL; MIC: 16 μg/mL) and *E. faecium* (IC_50_: 8 μg/mL; MIC: 32 μg/mL). Although representing a mixture of hundreds of secondary plant metabolites, dietE017a surprisingly reached a similar level of antibiotic activity exhibited in vitro by the single compound positive controls, namely chloramphenicol (*S. aureus*, IC_50_: 4 μg/mL; MIC: 32 μg/mL) and vancomycin (*E. faecium*, IC_50_: 4 μg/mL; MIC: 4 μg/mL). Extract dietE017a exhibited no cytotoxic effects in the human keratinocyte cell line (Cytotoxicity IC_50_: > 515 μg/mL). The calculated therapeutic index (TI) demonstrated that cytotoxicity to human cells was at concentrations > 128 (*S. aureus*) and > 64 (*E. faecium)* times higher than that required for growth inhibition of these pathogenic bacteria. In the Greater Mpigi region, none of the informants stated that *Z. chalybeum* is used as an herbal drug for skin infections. Instead, 18% of the traditional healers interviewed stated that this deciduous shrub or tree is used for wound disinfection and treatment. It was also reported that it is used medicinally for treatment of stomach/GI tract disorders (13%), nausea (8%) and sore throat (8%)^24^. These results support the traditional use of *Z. chalybeum* stem bark as an anti-infective therapy. *Z. chalybeum* has been moderately studied in the past. The majority of publications documented its traditional use in Uganda^24,39–42^, Kenya^43^, Tanzania^44^ and Ethiopia^45^. Fagaramide, an antiplasmodial natural product, was previously isolated from *Z. chalybeum* stem bark^46^. In contrast to our results, diverse extracts of stem bark did not show any antibacterial activity against *S. aureus* up to a concentration of 100 mg/mL in two other studies published in 2001 and 2011^47,48^. The present work offers the first report of antibiotic properties of *Z. chalybeum* stem bark against growth of multiresistant *S. aureus* and *E. faecium*.

Another highly active extract in terms of growth inhibition was an ethanolic extract of *H. madagascariensis* stem bark (etE011-18), displaying the same IC_50_ (8 μg/mL) and MIC (32 μg/mL) values against *E. faecium* EU-44 as *Z. chalybeum* (dietE017a)*.* Extract etE011-18 also highly inhibited growth of *S. aureus* UAMS-1 (IC_50_: 8 μg/mL; MIC: 32 μg/mL). Although there was low cytotoxicity against human keratinocytes recorded (IC_50_: 256 μg/mL), the TI still reached an excellent value of 32 for both strains. *H. madagascariensis* has been extensively studied in the past and reports on traditional medicine describe medicinal use all over the African continent^49–54^. One study sought to evaluate the antibacterial activity of stem bark from *H. madagascariensis* against bacterial species also tested in our study^55^. In this study, a hydro-ethanolic extract displayed low inhibitory effects on two *P. aeruginosa* strains (MIC: 500 μg/mL) and moderate effects on two *S. aureus* strains (MIC: 62.5 μg/mL and 125 μg/mL). Although much work has been done on this evergreen shrub or tree, including isolation of compounds^56–59^, our study is the first to report on the strong growth inhibitory activity of the stem bark of this species, targeting multiresistant ESKAPE pathogens, especially *E. faecium* and *S. aureus*. This finding strongly supports the traditional medicinal use of *H. madagascariensis* in the Greater Mpigi region, where it was highly cited to be efficient in treatment of skin infections (relative frequency of citation: 31%, *n* = 39); wound treatment (31%); stomach/gastrointestinal (GI) tract disorders (21%); nausea (13%), sore throat (13%); and fever (18%)^24^.

As stated above, the stem barks of *Z. chalybeum* and *H. madagascariensis* are particularly often used in plant-based antibiotic treatment of stomach/GI tract disorders and wounds in the Greater Mpigi region. Our study identified certain extracts of these stem barks as being highly effective in inhibiting bacterial growth of multidrug-resistant strains of *E. faecium* and *S. aureus*. Enterococci are mostly commensal non-pathogenic bacteria, present in the GI tract without causing human infections^60^. However, in past decades, *E. faecium* strains have emerged as one of the most pervasive nosocomial pathogens worldwide that caused numerous outbreaks of serious infections^61,62^. *E. faecium* managed to circumvent conventional antibiotics, such as vancomycin, and successfully adapted to hospital environments, making it difficult to target pharmacologically^63,64^. With regards to its prevalence in Africa, regional pathogenic strains of *E. faecium* were identified to possess the lowest vancomycin resistance rates worldwide, but at the same time the highest resistance to ampicillin (data provide by WHO regional offices)^65^. This might be due to regional scarcity and high prices for wide-spectrum antibiotics and higher prescription of narrow-spectrum antibiotics^66^. The traditional use of *Z. chalybeum* and *H. madagascariensis* is still widely practiced for treatment of stomach/GI disorders in our study region and specific use against *E. faecium* was therefore validated in this study. *S. aureus* is a ubiquitous colonizer of the human epithelia, e.g. the skin, the upper respiratory tract and the GI tract^67^. Methicillin-resistant *S. aureus* (MRSA) can cause serious, sometimes fatal, infections upon invading the blood-stream or internal tissues, whereas wounds are often the source of infection^68,69^. According to the results of our study, the traditional use of *Z. chalybeum* and *H. madagascariensis* stem barks in wound treatment and disinfection therefore seems justified in order to prevent and combat a *S. aureus* infection, among others.

None of the extracts reached an MIC in the growth inhibition experiments with *K. pneumoniae*, *A. baumannii* and *E. cloacae* (maximum concentration tested at 256 µg/mL) and the lowest IC_50_ values reported were 256 μg/mL for *K. pneumoniae* (dietE010, *T. asiatica*) and 32 μg/mL for *A. baumannii* (etE015, *C. molle*). Moreover, none of the 86 extracts were showed antibacterial effects on the growth of the multiresistant *E. cloacae* CDC-0032 strain.

Another set of extracts displayed antivirulence activity and was highly effective in the quorum sensing inhibition and the δ-toxin production screen. Selectively inhibiting quorum sensing pathways could prove to be an efficient alternative to antibiotics that simply try to kill the pathogen. One advantage of targeting the *agr* system is disruption of a wide variety of virulence factors, instead of targeting each virulence factor individually^70,71^. Use of botanical formulations or small molecule quorum sensing inhibitors isolated from medicinal plants might offer some additional benefits, e.g. protection of commensal bacteria that induce protective responses to prevent invasion and colonization by pathogens as part of the human host defense^70,72,73^. Many virulence factors are not of relevance to the overall survival of the pathogen. QSI therefore provides a less selective pressure towards resistance, facilitating a promising alternative therapy when combatting pathogens that are likely to develop resistance mechanisms during strong selective pressure of conventional treatment with antibiotics^73–75^.

The ethyl acetate root extract of *S. aculeastrum* (eE006) was among the two most QSI-active extracts, showing reporter gene subtype-dependent IC_50_ values of 4, 1, 16 and 64 μg/mL (*agr* I-IV). Its antivirulence activity was successfully confirmed in the δ-toxin production screen, where it significantly attenuated δ-toxin biosynthesis in our high-toxin-producing model strains. Extract eE006 exhibited no cytotoxicity in our model at the highest tested concentration of 512 μg/mL and calculated TIs were as high as > 128 (*agr* I)., > 512 (*agr* II)., > 32 (*agr* III). and > 8 (*agr* IV). *S. aculeastrum* is regarded an understudied species and our study accomplished to identify the roots of *S. aculeastrum* as strong quorum sensing inhibitor for the first time. Previous publications encompass use in African folklore medicine^24,76–79^. Pharmacological studies published on this species investigated the fruits or leaves, but not the roots^80–84^. For instance, fruits and leaves were investigated for antimicrobial activity against food-borne pathogens, but MIC values were only in the mg/mL range^85^. Steroidal alkaloids have previously been isolated from the root bark and fruits, such as solaculine A^86^ which induced induces non-selective cytotoxicity and P-glycoprotein inhibition^87^. At our field study location, the Greater Mpigi region, this poisonous nightshade species, whose berries contain α-solanine^88^, is often used in disinfection and treatment of wounds (23%) and fever (15%)^24^. The pharmacological effects of the roots, claimed by the traditional healers, might be explained by its now reported antivirulence activity, but should be further investigated through additional studies.

The hexane extract of *S. calycinum *subsp*. angustifolium* leaves (hE004-18) also exhibited strong antivirulence effects. Quorum sensing inhibition IC_50_ values against *agr* I–IV were as low as 4, 2, 16 and 32 μg/mL. No cytotoxicity was found, suggesting that use of this plant extract and species is safe to human cells. Calculated TIs are reported as > 128 (*agr* I), > 256 (*agr* II), > 32 (*agr* III) and > 16 (*agr* IV). *S. calycinum subsp. angustifolium* is still a highly understudied species with only five studies previously being published on its traditional medicinal use^24,89–92^ and one study that reported the presence of the hydrocarbon nonacosane and the glucosinolate, glucoiberverin^93^ in its leaves. Traditional healers in the Greater Mpigi region claimed that the leaves of this medicinal herb are often used to treat skin infections (15%), wounds (26%), disorders of the stomach/GI tract (13%), sore throats (10%) and fever (10%)^24^. Our study provides the first report of antivirulence activity, targeting quorum sensing and δ-toxin production in *S. aureus*, validating *S. calycinum subsp. angustifolium* application as anti-infective herbal drug.

Furthermore, our quorum sensing inhibition experiments showed that eE006 and hE004-18 displayed lower IC_50_ and MIC values than the positive control (224CF2c). This is particularly interesting because 224CF2c is not a crude extract, but a refined fraction of the European chestnut (*Castanea sativa*) that was previously identified to be highly active against *agr* I–IV^14^. Future bioassay-guided fractionation of the crude extracts eE006 and hE004-18 from the Ugandan rainforests could result in promising novel natural products aiming towards discovery of antivirulence drugs. The traditional use of *S. aculeastrum* roots (eE006) and *S. calycinum subsp. angustifolium* leaves (hE004-18) in wound treatment^24^ indicates that these extracts might demonstrate a significant reduction in dermonecrosis after infection with a virulent strain of MRSA, as shown with QSI-active fractions from *Schinus terebinthifolia* (Brazilian Peppertree) and *Castanea sativa* (European Chestnut) before^13,14^. Moreover, it will be essential to further investigate the ability of eE006 and hE004-18 in limiting the severity of disease and in increasing efficacy of conventional antibiotics. This includes potential activation of other virulence pathways, such as biofilm formation and secretion systems. Further experiments are also needed in order to assess the actual decrease of S*. aureus* virulence in vivo*.*

## Methods

### Ethnobotanical data

Information on traditional use for medical treatment among 39 traditional healers in the Greater Mpigi region in Uganda was obtained by means of an ethnobotanical survey. Results of this study were previously published^24^ and serve as a basis for the antibacterial and antivirulence experiments.

### Collection and identification of plant material

Plant specimens were collected under guidance of the traditional healers during fieldwork in 2015, 2016 and 2017, while following standard collection procedures^94^. The approach for plant identification and assignment of scientific names was adapted from Weckerle et al. ^95^. Scientific names were cross-checked with https://www.theplantlist.org. Plant family assignments follow The Angiosperm Phylogeny Group IV guidance^96^. Voucher specimens of all species collected were deposited at Makerere University Herbarium in Kampala, Uganda and select specimens were also deposited at the Emory University Herbarium (GEO) in Atlanta, GA, USA and made digitally available on the SERNEC portal^97^ (Supplementary Table S1).

### Extraction

Plant samples were shade dried and ground prior to extraction (Supplementary Figure S1). Extractions were performed as described in the flow sheet (Supplementary Figure S2). Briefly, plant material was either extracted by maceration, Soxhlet extraction or aqueous decoction. In order to selectively extract different compounds from the samples, extraction procedures were conducted using solvents of different polarities. Some plant species were collected for a second time in order to facilitate for production of higher amounts of extract. These upscaled extractions were performed in 2018 and resulting extracts received the additional information “-18” in their extract ID.

### Bacterial strains

Multidrug-resistant clinical isolates were used in all growth-inhibition experiments in order to realistically assess the results of this study for future drug discovery advances for AMR threats. This study used 12 strains from six bacterial species recognized as ESKAPE pathogens, including Gram-negative [*Klebsiella pneumoniae* (CDC-004), *Acinetobacter baumannii* (CDC-0033), *Pseudomonas aeruginosa* (AH-71) and *Enterobacter cloacae* (CDC-0032)] and Gram-positive [*Enterococcus faecium* (EU-44) and *Staphylococcus aureus* (UAMS-1, AH-1677, AH-430, AH-1747, AH-1872, AH-1872, NRS243)] species. Strain characteristics, antibiotic resistance profiles and sources are reported in Supplementary Table S2. After streaking from freezer stock and overnight incubation at 37 °C, all strains were maintained on tryptic soy agar (TSA). Overnight liquid cultures were achieved in tryptic soy broth (TSB) at 37 °C and with constant shaking at 230 rpm. Appropriate positive controls (antibiotics or quorum quenchers) and negative controls (vehicle control, sterile media control) were always incorporated into the assays. All bacterial experiments were conducted in triplicate and repeated at least once on a separate day.

### Growth inhibition assay

All growth inhibition experiments were conducted following the guidelines set by the Clinical and Laboratory Standards Institute for broth microdilution testing^98^. Standardized working cultures were calculated and diluted from TSB overnight cultures in cation-adjusted Müller-Hinton broth (CAMHB). This was achieved using a BioTek Cytation3 and based on the cultures’ optical density (OD_590 nm_) to a confluence of 5 × 10^5^ CFU/mL. The working culture was pipetted into 96-well microtiter plates (Greiner Bio-One International, CELLSTAR 655–185) and extracts and controls were added. Vehicle controls, sterility controls and antibiotic controls (1–64 µg/mL) were included on the plate setup. After initial optical density readings at 600 nm to account for extract absorbance, plates were incubated at 37 °C for 18 h (*E. faecium*, *S. aureus*, *P. aeruginosa*, *E. cloacae*) or for 22 h (*A. baumannii*, *K. pneumoniae*). A final optical density measurement was performed, and the percent inhibition was calculated as previously described^99^. Growth inhibition is reported as the IC_50_ (the lowest concentration at which a sample displayed ≥ 50% inhibition) and MIC (the lowest concentration at which a sample displayed ≥ 90% inhibition).

All extracts were tested at a concentration of 256 µg/mL during an initial screen. Extracts that displayed a percent inhibition above 40% for an individual strain were further examined by dose-response experiments to obtain the IC_50_ and MIC values. Using two-fold serial dilution, extracts and vehicle were tested at concentrations ranging from 2 to 256 µg/mL.

### *agr* reporter assay

An initial library screen was first conducted against the *agr* I reporter strain of *S. aureus* at 16 μg/mL (sub-MIC concentrations). After identification of extracts that displayed > 40% inhibition, candidates were further examined by dose-response studies (0.5–64 μg/mL) against all four accessory gene regulator (*agr*) subtypes of *S. aureus*. Crude extracts were tested as previously described^14,100^. Briefly, the *agr* reporter strains were grown and maintained in TSB and TSA, supplemented with chloramphenicol (10 μg/mL). All *agr* inhibition assays were conducted in 96-well, tissue culture-treated, black-sided microtiter plates (Costar 3,603, final well volume: 200 μL). Microtiter plates were incubated in a humidified chamber at 37 °C, while shaking at 1,200 rpm (Stuart SI505). At initial (0 h) and final (22 h) time points, OD_600nm_ and fluorescence (493 nm excitation, 535 nm emission) were measured using a plate reader (BioTek Cytation3). Controls included a vehicle control (DMSO) and a positive control (224CF2c). 224CF2c is an QSI-active fraction extracted from the European chestnut (*Castanea sativa*), as reported in a previous study by the authors^14^. The quorum-quenching activity was reported as percent vehicle of the signal of the individual reporter train’s yellow fluorescent protein (YFP). Dose–response curves were generated using the GraphPad Prism 8 software (GraphPad Software, La Jolla, CA, USA). IC_50_ and MIC values were calculated as in the growth inhibition experiments described above.

### Production of δ-toxin and quantification by HIC-HPLC

To confirm antivirulence activity on the translational products of *agr* in *S. aureus* (decline in δ-toxin biosynthesis), the most active extracts of the *agr* reporter assay were tested at 8, 16, and 32 μg/mL and compared to an untreated control in a δ-toxin production assay using high-toxin-producing strains of *S. aureus* (AH1262 and NRS243) by hydrophobic interaction chromatography as previously described^37^. The positive control 224C-F2c was additionally tested at 64 μg/mL. Data integration was normalized for growth (OD_600nm_) during supernatant harvest and reported as formylated and deformylated δ-toxin, visualized by a stacked histogram chart. The data was analyzed using a Student's t-test and statistical significance was denoted as *P value < 0.05, ^‡^*P* value < 0.01, ^†^*P* value < 0.001.

### Human keratinocyte toxicity assay

Potential cytotoxicity of extracts was assessed using immortalized human keratinocytes (HaCaTs cells) combined with a lactate dehydrogenase (LDH) test kit (G-Biosciences, St. Louis, MO, USA) as previously described by Quave et al.^14^. All extracts were initially tested at a concentration of 64 μg/mL. In effort to calculate the therapeutic index (TI), samples selected for dose-response cytotoxicity testing were extracts that (a) displayed cytotoxic activity above 50% inhibition in the library screen at 64 μg/mL, (b) were introduced as library-screen-active candidates to the growth inhibition dose-response studies and (c) were identified as being most active in the quorum sensing-dose-response studies and were investigated further in the δ-toxin production and quantification assay. Dose-response cytotoxicity experiments were conducted at a concentration range of 2–256 μg/mL. Percent of the vehicle (DMSO, v/v) in the well was < 2% for all experiments. All human keratinocyte toxicity experiments were conducted in triplicate and repeated at least once on a separate day. The TI for growth inhibition and the TI for quorum-sensing inhibition (*agr* I-IV) were calculated by dividing the IC_50_ for extract cytotoxicity by the IC_50_ for its respective antibacterial activity.

### LC–MS characterization of extracts

Extracts displaying the highest anti-growth and antivirulence activity in the in vitro assays, as well as the highest TI, were selected for chemical characterization. These extracts were examined by negative ESI mode Liquid chromatography-Fourier transform mass spectrometry (LC-FTMS) using a Thermo Scientific LTQ-FT Ultra mass spectrometer equipped with a Shimadzu SIL-ACHT auto sampler and Dionex 3600SD HPLC pump. A 20 μL injection of the extract at 10 mg/mL dissolved in ethyl acetate, 1:1 ethyl acetate:methanol, or DMSO was made onto a Phenomenex Kinetex C18 150 × 2.1 mm, 2.6 µm with compatible guard column at room temperature. The mobile phase consisted of (A) 0.1% formic acid in water and (B) 0.1% formic acid in Optima LC/MS acetonitrile (Fisher Scientific) at a flow rate of 0.2 mL/min. The gradient program began with initial conditions of 98:2 A:B, which were held for 3 min, then changed to 100% B over 15 min using a linear gradient, 100% B was held for 25 min, before returning to initial conditions to equilibrate the column. The capillary temperature and voltage were 275.0 °C and − 48.00, the sheath gas flow 40, source voltage and current − 5.0 kV and 100.0 μA. All MS data was collected in negative MS^1^ mode scanning from *m/z* 150–1,500 with data dependent MS^2^ collected on the top 4 most abundant ions. The data was collected and processed using Thermo Scientific Xcalibur 2.2 SP1.48 software.

Putative compounds for each extract were determined by searching The Dictionary of Natural Products (CRC Press) and Scifinder (Chemical Abstracts Service) for compounds consistent with each MS^1^ peak’s parent ion *m/z* (± 1 Da). For The Dictionary of Natural Products ions were searched against all compound records for the extract’s genus. During searches in Scifinder, ions were screened against compounds published from the same genus as the extract in books, clinical trials, commentaries, conference proceedings, dissertations, editorials, journals, letters, reports, and review articles; entries from patents and preprints were not included in the search. Any matches from these databases were compared to the empirical formulas derived from the experimental MS data. Compounds which matched the empirical formula with a calculated mass error < 10 ppm were investigated further in the literature and reported. When no matches in the literature were found, the hydrocarbon with the lowest mass error was reported. Due to search limitations in Scifinder, only compounds published prior to 2005 were searched for the genus *Solanum*. All searches were performed in Feb. 2020.

## Supplementary information

Supplementary file 1 (PDF 1211 kb)
