# Supplementary material for: Targeting ESKAPE pathogens with anti-infective medicinal plants from the Greater Mpigi region in Uganda
Source: Sci Rep. 2020 Jul 20;10:11935. doi: 10.1038/s41598-020-67572-8 (PMC7371678; doi:10.1038/s41598-020-67572-8)
Supplement: Supplementary file 1 — (PDF 1211 kb) [file 41598_2020_67572_MOESM1_ESM.pdf]

Targeting ESKAPE pathogens with anti-infective medicinal plants from the Greater Mpigi region in Uganda

Fabien Schultz[a,b,c], Godwin Anywar[d], Huaqiao Tang[f], François Chassagne[f], James T. Lyles[f], Leif-Alexander Garbe[b,c,e], Cassandra L. Quave[a,f,g*]

[a]*Department of Dermatology, Emory University School of Medicine, 615 Michael St., Atlanta, 30322, Georgia, United States of America*
[b]*Institute of Biotechnology, Faculty III - Process Sciences, Technical University of Berlin, Gustav-Meyer-Allee 25, Berlin, 13355, Germany*
[c]*Department of Agriculture and Food Sciences, Neubrandenburg University of Applied Sciences, Brodaer Str. 2, Neubrandenburg, 17033, Germany*
[d]*Department of Plant Sciences, Microbiology and Biotechnology, Makerere University, P.O Box 7062,Kampala, Uganda*
[e]*ZELT - Neubrandenburg Center for Nutrition and Food Technology gGmbH, Seestraße 7A, Neubrandenburg, 17033, Germany*
[f]*Center for Study of Human Health, Emory University College of Arts and Sciences, 615 Michael St., Atlanta, 30322, Georgia, United States of America*
[g]*Emory Antibiotic Resistance Center, Emory University, 615 Michael St., Atlanta, 30322, Georgia, United States of America*

*Corresponding author (*Email address:* cquave@emory.edu)

Supplementary information

Table of contents

Supplementary Figure S1:
Photos of dried plant material page 3

Supplementary Figure S2:
Workflow of extractions page 4

Supplementary Table S1:
Description of collected plant species page 5

Supplementary Table S2:
Description of bacterial strains used in the study page 7

Supplementary Table S3:
Results of HaCaT cytotoxicity library screen at 64 μg/mL page 9

Supplementary Figure S3:
Chemical structures for the putative matches from the extract of *H. madagascariensis*, etE011-18 page 12

Supplementary Figure S4: Chemical structures for the putative matches from the extract of *S. calycinum* subsp. *angustifolium,* hE004-18 page 13

Supplementary Figure S5:
Chemical structures for the putative matches from the extract of *S. aculeastrum*, eE006 page 14

Supplementary Figure S6: Chemical structures for the putative matches from the extract *Z. chalybeum,* dietE017a page 15

References cited in supplementary files page 15

Supplementary Figure S1: Photos of dried plant material

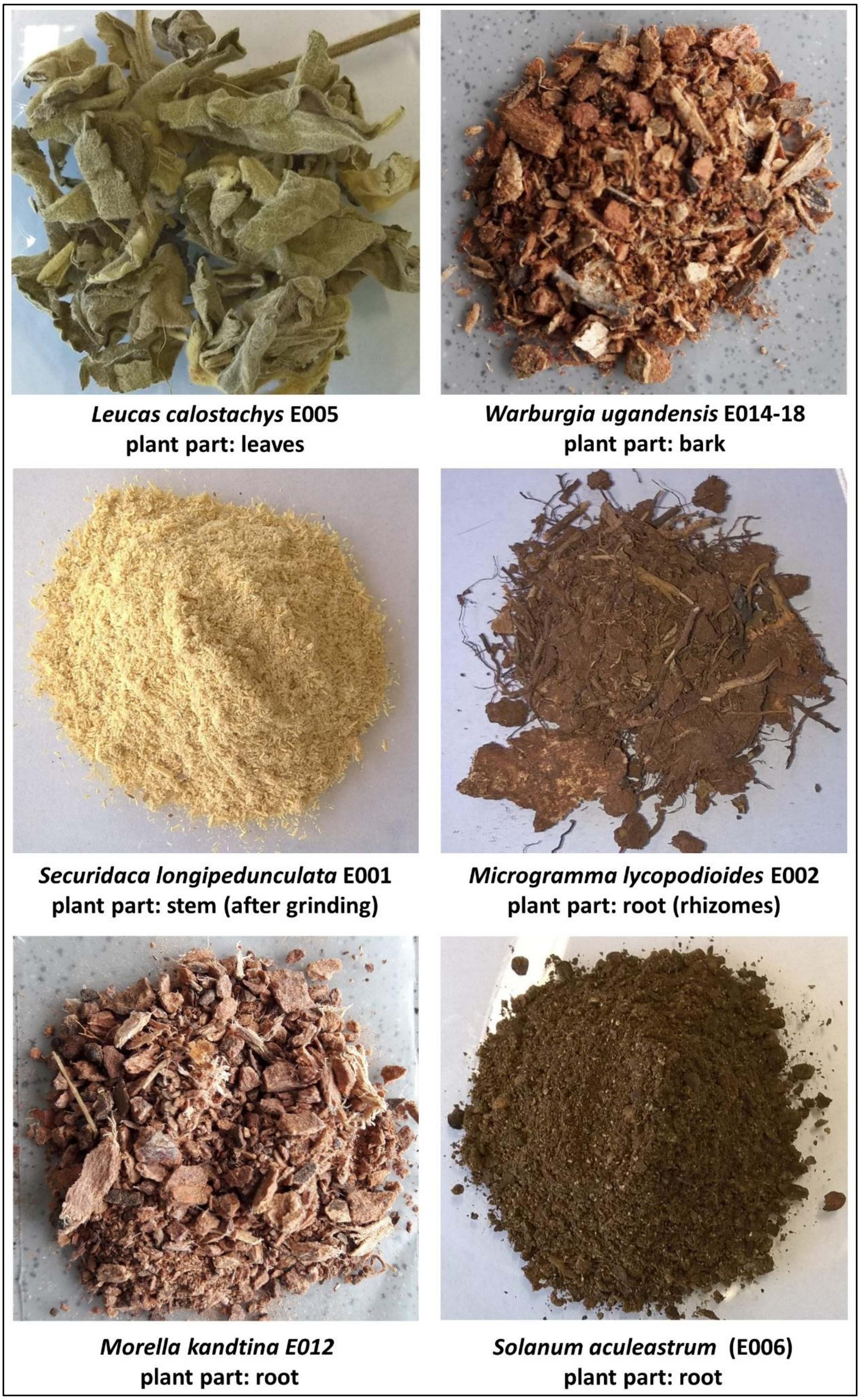

Supplementary Figure S2: Workflow of extractions

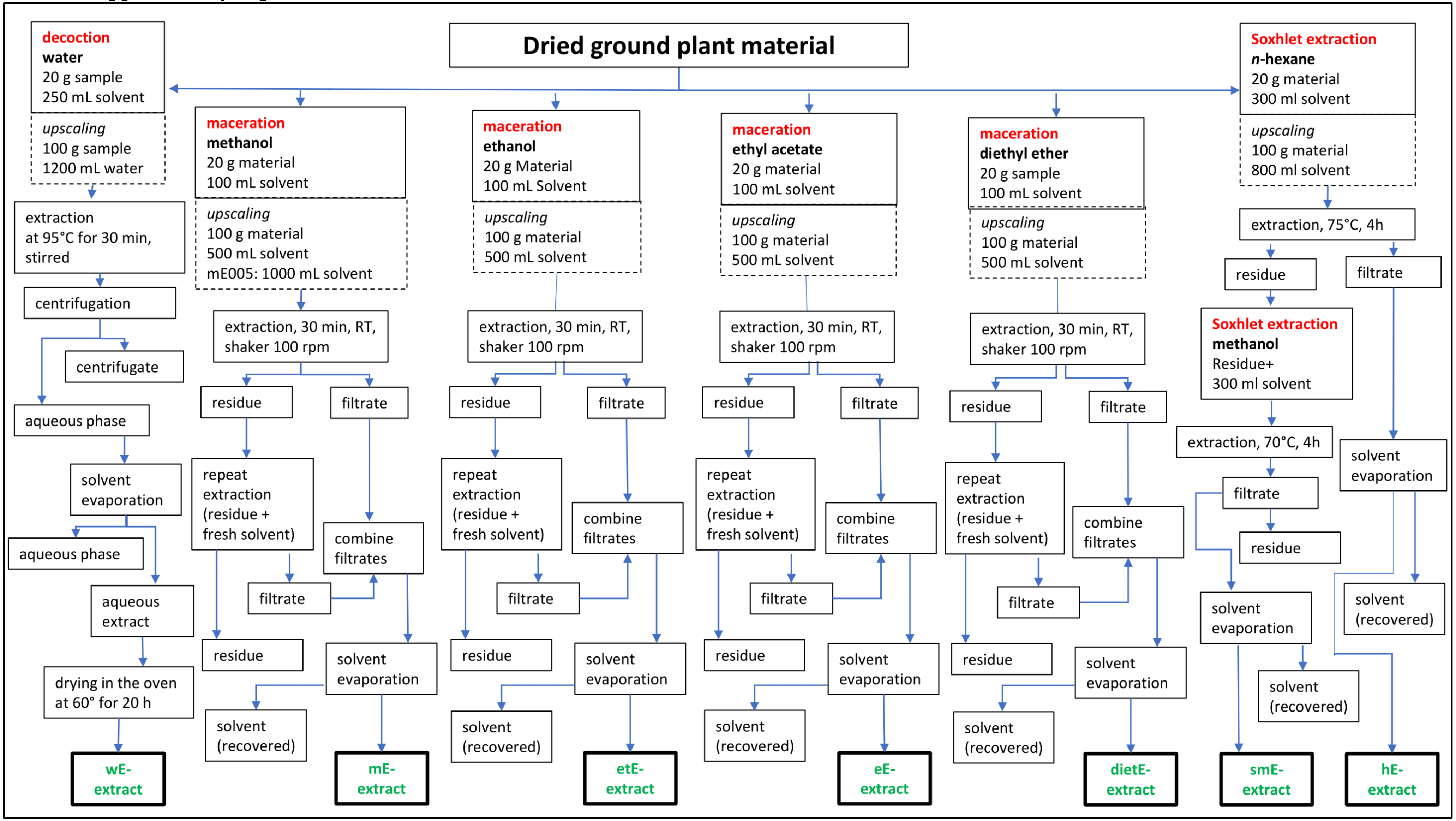

Supplementary Table S1: Description of collected plant species. All specimens deposited at Emory University have been digitized and are available for viewing at http://sernecportal.org/portal/.

Extract ID	Type of extract	Scientific name	Local name in Luganda	Plant part	Voucher specimen #
**eE001**	ethyl acetate	*Securidaca longipedunculata* Fresen., Polygalaceae	Mukondwe	stem	AG196°
**wE001**	water				
**hE001**	hexane (sox.)				
**mE001**	methanol				
**smE001**	methanol (sox. succ.)				
**eE002**	ethyl acetate	*Microgramma lycopodioides* (L.) Copel., Polypodiaceae	Kukumba	root (rhizomes)	AG639°
**wE002**	water				
**hE002**	hexane (sox.)				
**mE002**	methanol				
**smE002**	methanol (sox. succ.)				
**eE003**	ethyl acetate	*Ficus saussureana* DC., Moraceae	Muwo	stem	AG219°
**wE003**	water				
**hE003**	hexane (sox.)				
**mE003**	methanol				
**smE003**	methanol (sox. succ.)				
**eE004**	ethyl acetate	*Sesamum calycinum subsp. angustifolium* (Oliv.) Ihlenf. & Seidenst., Pedaliaceae	Lutungotungo	leaves	AG205° 23173*
**wE004**	water				
**hE004**	hexane (sox.)				
**mE004**	methanol				
**smE004**	methanol (sox. succ.)				
**eE004-18**	ethyl acetate				
**hE004-18**	hexane (sox.)				
**smE004-18**	methanol (sox. succ.)				
**eE005**	ethyl acetate	*Leucas calostachys* Oliv., Lamiaceae	Kakuba musulo	leaves	AG195° 23175*
**wE005**	water				
**hE005**	hexane (sox.)				
**smE005**	methanol (sox. succ.)				
**eE005-18**	ethyl acetate				
**hE005-18**	hexane (sox.)				
**mE005-18**	methanol				
**smE005-18**	methanol (sox. succ.)				
**eE006**	ethyl acetate	*Solanum aculeastrum* Dunal, Solanaceae	Kitengo	root	AG193°
**wE006**	water				
**hE006**	hexane (sox.)				

**smE006**	methanol (sox. succ.)				
**eE007**	ethyl acetate	*Albizia coriaria* Oliv., Fabaceae	Mugavu	bark	AG203°
**etE007**	ethanol				
**eE008**	ethyl acetate	*Erythrina abyssinica* DC., Fabaceae	Jjirikiti	bark	AG199°
**etE008**	ethanol				
**eE009**	ethyl acetate	*Zanthoxylum chalybeum* Engl., Rutaceae	Ntaleyaddungu	bark	AG204°
**etE009**	ethanol				
**eE010**	ethyl acetate	*Toddalia asiatica* (L.) Lam., Rutaceae	Kawule	leaves, bark	AG190°
**etE010**	ethanol				
**dietE010**	diethyl ether				
**etE010a**	ethanol				
**eE011**	ethyl acetate	*Harungana madagascariensis* Lam. ex Poir., Hypericaceae	Mukabiiransiko	bark	AG230° 23174*
**etE011**	ethanol				
**dietE011**	diethyl ether				
**etE011a**	ethanol				
**eE011-18**	ethyl acetate				
**wE011-18**	water				
**etE011-18**	ethanol				
**dietE011-18**	diethyl ether				
**hE011-18**	hexane (sox.)				
**smE011-18**	methanol (sox. succ.)				
**eE012**	ethyl acetate	*Morella kandtiana* (Engl.) Verdc. & Polhill, Myricaeae	Mukikimbo	root	AG201° 23174*
**etE012**	ethanol				
**dietE012**	diethyl ether				
**etE012a**	ethanol				
**eE012-18**	ethyl acetate				
**wE012-18**	water				
**etE012-18a**	ethanol				
**etE012-18b**	ethanol				
**dietE012-18**	diethyl ether				
**eE013**	ethyl acetate	*Cassine buchananii* Loes., Celastraceae	Mbaluka	bark	AG198°
**etE013**	ethanol				
**etE013a**	ethanol				
**eE014**	ethyl acetate	*Warburgia ugandensis* Sprague, Canellaceae	Abasi	bark	AG220° 23181*
**etE014**	ethanol				
**dietE014**	diethyl ether				
**etE014a**	ethanol				
**eE014-18**	ethyl acetate				
**wE014-18**	water				
**etE014-18**	ethanol				
**dietE014-18**	diethyl ether				
**hE014-18**	hexane (sox.)				
**smE014-18**	methanol (sox. succ.)				

**eE015**	ethyl acetate	*Combretum molle* R.Br. ex G.Don, Combretaceae	Ndagi	bark	AG191°
**etE015**	ethanol				
**dietE016**	diethyl ether	*Plectranthus hadiensis* (Forssk.) Schweinf. ex Sprenger, Lamiaceae	Kibwankulata	leaves	AG210°
**hE016**	hexane				
**etE017**	ethanol	*Zanthoxylum chalybeum* Engl., Rutaceae	Ntaleyaddungu	bark	AG204°
**dietE017**	diethyl ether				
**etE017a**	ethanol				
**dietE017a**	diethyl ether				

° deposited at Makerere University herbarium   * deposited at Emory University herbarium

Abbreviations: sox.: Soxhlet extraction; succ.: successive extraction

Supplementary Table S2: Description of bacterial strains used in the study

Species	Strain ID	Characteristics*	Ref.
***E**nterococcus faecium*	EU-44	HM-959; Strain 513<br>Resistance: AMC, RIF, SXT, TET, TZP<br>Human clinical sample, source: BEI Resources	
***S**taphylococcus aureus*	UAMS-1	ATCC49230<br>Clinical MSSA isolate from osteomyelitis<br>Source: Dr. Mark Smeltzer, University of Arkansas for Medical Sciences	1
	AH-1677	AH845 + pDB59 cmR*;* Resistance: OXA<br>*agr* type I YFP reporter<br>Source: Dr. Alex Horsewill, UC Denver	2
	AH-430	SA502a + pDB59 cmR*;*<br>*agr* type II YFP reporter<br>Source: Dr. Alex Horsewill, UC Denver	2
	AH-1747	MW2 + pDB59 cmR*;* Resistance: OXA<br>*agr* type III YFP reporter<br>Source: Dr. Alex Horsewill, UC Denver	2
	AH-1872	MN EV(AH407) + pDB59 cmR<br>*agr* type IV YFP reporter<br>Source: Dr. Alex Horsewill, UC Denver	2
	NRS243	HT20020252<br>Resistance: ERY, PEN<br>Intermediate resistance: CIP<br>high delta toxin producing strain associated with pneumonia<br>Source: NARSA Library	
	AH1263	LAC CA-MRSA USA300 clinical isolate<br>Resistance: OXA<br>high delta toxin producing strain<br>Source: Source: Dr. Alex Horsewill, UC Denver	
***K**lebsiella pneumoniae*	CDC-004	AR-BANK#0004<br>Resistance: AMC, AMP, ATM, CAZ, CFZ, CIP,	

		CRO, CTX, DOR, ETP, FEP, FOX, IPM, LVX, MEM, SAM, SXT, TET, TOB, TZP<br>Clinical isolate, source: CDC Antimicrobial Resistance Bank	
***A**cinetobacter baumannii*	CDC-0033	AR-BANK #0033<br>Resistance: CAZ, CIP, CLI, CRO, CTX. DOR, FEP, GEN, IPM, LVX, MEM, SAM, SXT, TOB, TZP<br>Clinical isolate, source: CDC Antimicrobial Resistance Bank	
***P**seudomonas aeruginosa*	AH-71	PAO1<br>Laboratory strain, source: Dr. Alex Horswill, University of Colorado, Denver	
***E**nterobacter cloacae*	CDC-0032	AR-BANK #0032<br>Resistance: AMC, AMP, ATM, CAZ, CFZ, CRO, CTX, ETP, FEP, FOX, GEN, IPM, MEM, SAM, SXT, TZP<br>Intermediate resistance: DOR, TOP<br>Clinical isolate, source: CDC Antimicrobial Resistance Bank	

***abbreviations:**

agr: accessory gene regulator; AMC: amoxicillin-clavulanic acid; AMP: ampicillin; ATM: aztreonam; CAZ: ceftazidime; CFZ: cefazolin; CIP: ciprofloxacin; CLI: clindamycin; CRO: ceftriaxone; cmR: Chloramphenicol resistance protein; CTX: cefotaxime; DOR: doripenem; ERY: erythromycin; ETP: ertapenem; FEP: cefoxitin; FOX: cefoxitin; GEN: gentamicin; IPM: imipenem; LVX: levofloxacin; MEM: meropenem; MSSA: methicillin sensitive *Staphylococcus aureus*; NARSA: network on antibiotic resistant *Staphylococcus* aureus; OXA: oxacillin; PEN: penicillin; RIF: rifampicin; SAM: ampicillin-sulbactam; SXT: trimethoprim-sulfamethoxazole; TET: tetracycline; TOB: tobramycin; TZP: piperacillin-tazobactam; YFP: yellow fluorescent protein

Supplementary Table S3: Results of HaCaT cytotoxicity library screen at 64 µg/mL

scientific name	extract ID	*HaCaT*	
		% cytoxoxicity ≥ 50	s
Securidaca longipedunculata	eE001	**negative**	-
	smE001	**negative**	-
	wE001	**negative**	-
	mE001	**negative**	-
	hE001	**negative**	-
Microgramma lycopodioides	hE002	**negative**	-
	mE002	**negative**	-
	wE002	**negative**	-
	smE002	**negative**	-
	eE002	**negative**	-
Ficus saussureana	smE003	**negative**	-
	wE003	**negative**	-
	eE003	**negative**	-
	mE003	**negative**	-
	hE003	**negative**	-
Sesamum calycinum subsp. angustifolium	smE004	**negative**	-
	smE004-18	**negative**	-
	mE004	**negative**	-
	hE004	**negative**	-
	hE004-18	**negative**	-
	eE004	**negative**	-
	eE004-18	**negative**	-
	wE004	**negative**	-
Leucas calostachys	eE005	**negative**	-
	eE005-18	**negative**	-
	smE005	**negative**	-
	smE005-18	**negative**	-
	wE005	**negative**	-
	mE005-18	**negative**	-
	hE005	**negative**	-
	hE005-18	**negative**	-
Solanum aculeastrum	eE006	**negative**	-
	hE006	**negative**	-
	wE006	**negative**	-
	smE006	**positive 51.8**	1.5

Albizia coriaria	etE007	**negative**	-
	eE007	**negative**	-
Erythrina abyssinica	etE008	**negative**	-
	eE008	**negative**	-
Zanthoxylum chalybeum	etE009	**negative**	-
	eE009	**negative**	-
	etE017	**negative**	-
	etE017a	**negative**	-
	dietE017	**negative**	-
	dietE017a	**negative**	-
Toddalia asiatica	etE010	**negative**	-
	etE010a	**negative**	-
	eE010	**negative**	-
	dietE010	**negative**	-
Harungana madagascariensis	etE011	**negative**	-
	etE011a	**negative**	-
	etE011-18	**negative**	-
	eE011	**negative**	-
	eE011-18	**negative**	-
	dietE011	**negative**	-
	dietE011-18	**negative**	-
	wE011-18	**negative**	-
	hE011-18	**negative**	-
	smE011-18	**negative**	-
Morella kantiana	etE012	**negative**	-
	etE012a	**negative**	-
	etE012-18a	**negative**	-
	etE012-18b	**negative**	-
	eE012-18	**negative**	-
	wE012-18	**negative**	-
	dietE012	**negative**	-
	dietE012-18	**negative**	-
Cassine buchananii	etE013	**negative**	-
	etE013a	**negative**	-
	eE013	**negative**	-
Warburgia ugandensis	dietE014	**negative**	-
	dietE014-18	**negative**	-
	eE014-18	**negative**	-
	wE014-18	**negative**	-
	hE014-18	**negative**	-
	smE014-18	**negative**	-

	etE014a	**negative**	-
	etE014-18	**negative**	-
Combretum molle	etE015	**negative**	-
	eE015	**negative**	-
Plectranthus hadiensis	hE016	**negative**	-
	dietE016	**negative**	-

Supplementary Figure S3: Chemical structures for the putative matches from the extract of *H. madagascariensis*, etE011-18

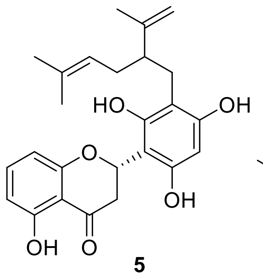

5

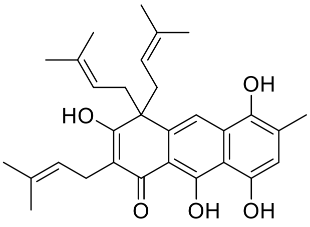

6a

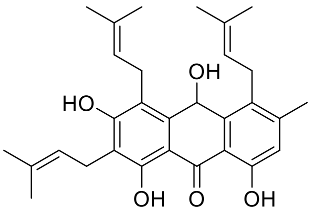

6b

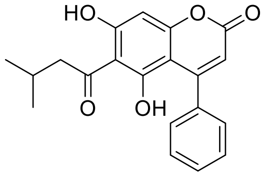

9a

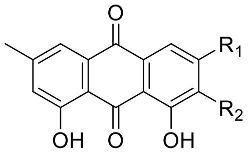

	R_1	R_2
**9b**	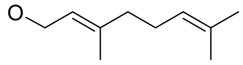	H
**9c**	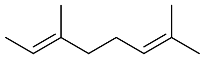	OH

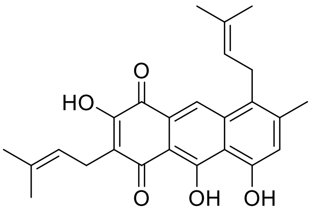

9d

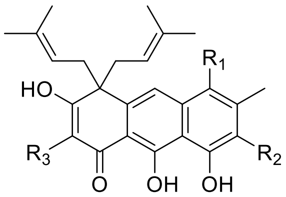

	12a	12c	12d
R_1	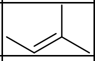	H	H
R_2	H	H	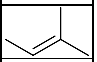
R_3	H	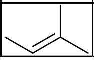	H

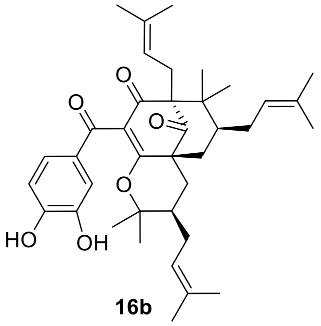

16b

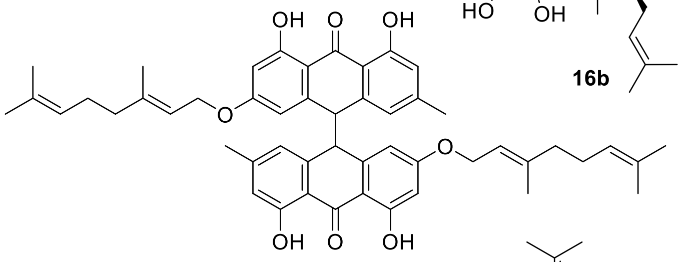

13

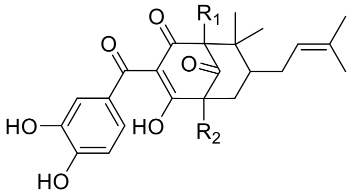

	R_1	R_2
**16a**	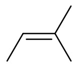	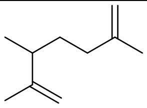
**16c & 16d**	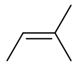	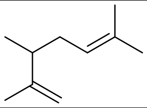
**16e**	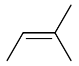	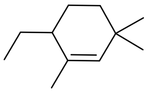
**16f**	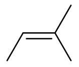	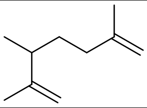
**16g**	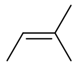	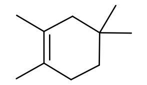

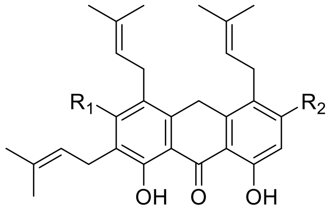

	R_1	R_2
**12b**	OH	CH_3
**12e**	CH_3	OH

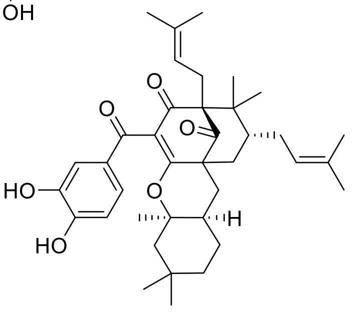

16h

Supplementary Figure S4: Chemical structures for the putative matches from the extract of *S. calycinum* subsp. *angustifolium,* hE004-18

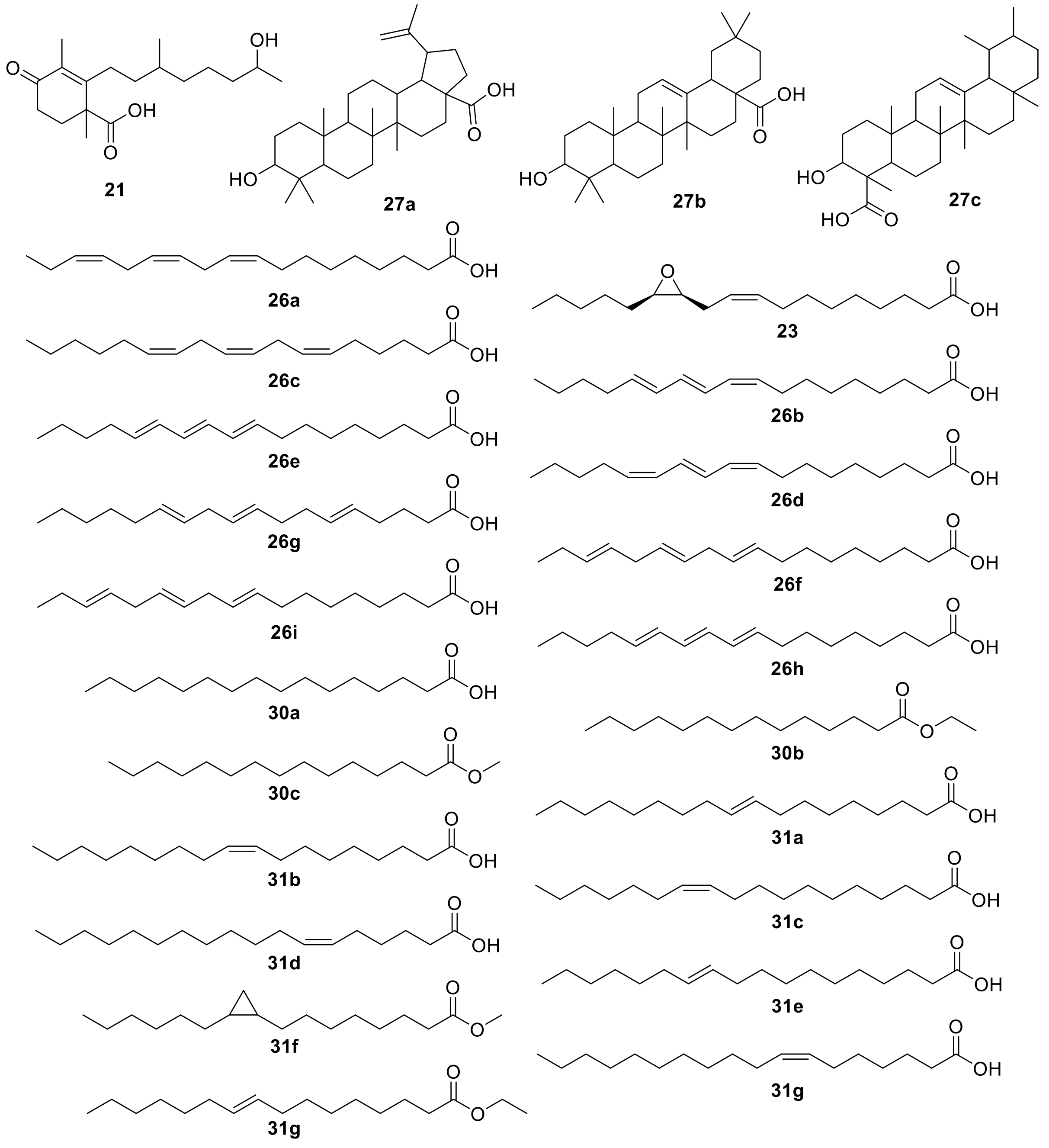

Supplementary Figure S5: Chemical structures for the putative matches from the extract of *S. aculeastrum*, eE006

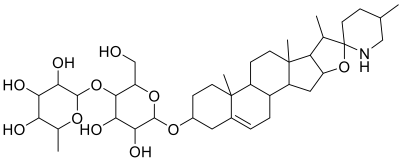

34a, 34c, 34h

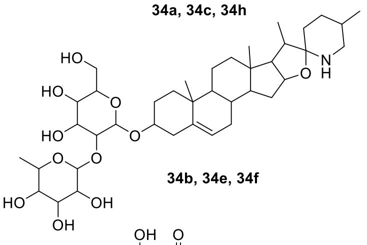

34b, 34e, 34f

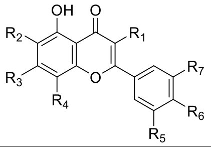

	R_1	R_2	R_3	R_4	R_5	R_6	R_7
**35a**	H	H	OH	H	H	OH	OMe
**35b**	H	H	OH	H	OH	OMe	H
**35c**	OH	H	OMe	H	H	OH	H
**35d**	H	OMe	OH	H	H	OH	H
**35e**	OMe	H	OH	H	H	OH	H
**35f**	H	H	OH	OH	H	OMe	H
**35g**	H	H	OMe	OH	H	OH	H

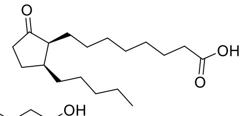

36b

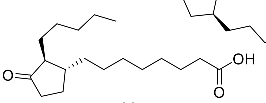

36a

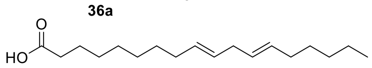

40

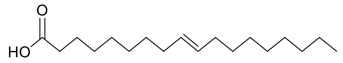

42

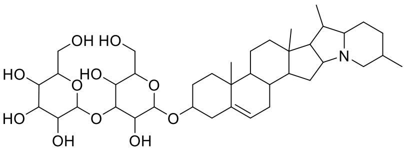

34d

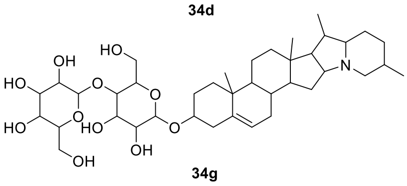

34g

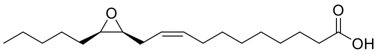

36c

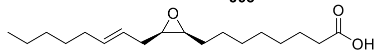

36d

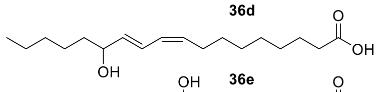

36e

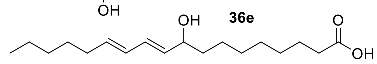

36f

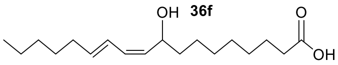

36g

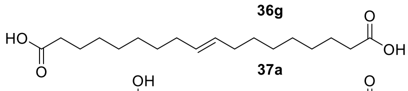

37a

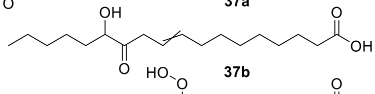

37b

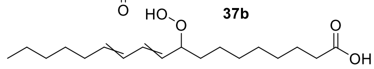

37c & 37 d

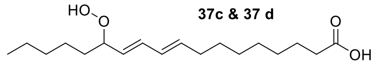

37e

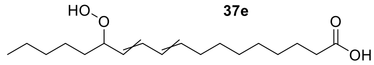

37f

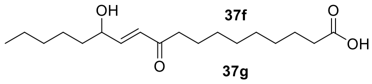

37g

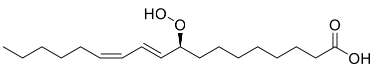

37h

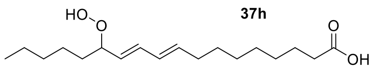

37i

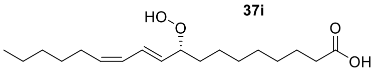

37j

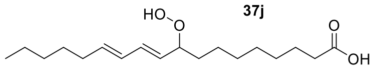

37k

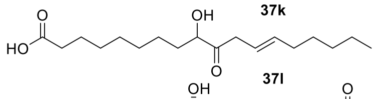

37l

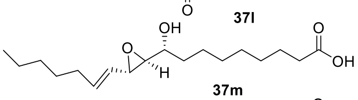

37m

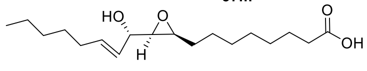

37n

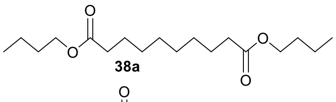

38a

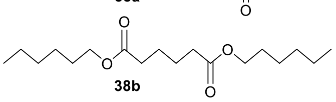

38b

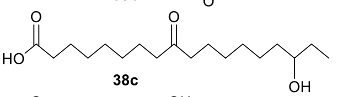

38c

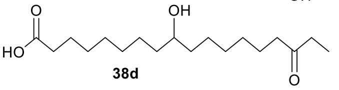

38d

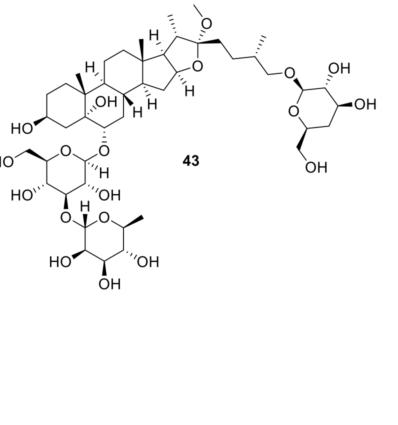

43

Supplementary Figure S6: Chemical structures for the putative matches from the extract *Z. chalybeum,* dietE017a

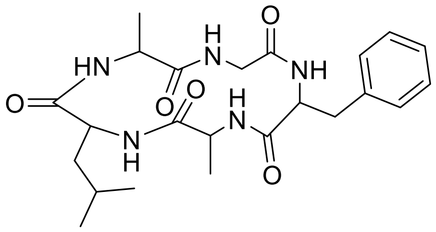

54

References cited in supplementary files

1 Gillaspy, A. F. *et al.* Role of the accessory gene regulator (agr) in pathogenesis of staphylococcal osteomyelitis. *Infect. Immun.* **63**, 3373-3380 (1995).

2 Kirchdoerfer, R. N. *et al.* Structural basis for ligand recognition and discrimination of a quorum-quenching antibody. *J. Biol. Chem.* **286**, 17351-17358, doi:10.1074/jbc.M111.231258 (2011).
